# Bis[3α,7α,12α-tris­(4-nitro­benzo­yloxy)-5β-cholan-24-yl] disulfide–ethyl acetate–*n*-hexane (4/4/1)

**DOI:** 10.1107/S1600536810050385

**Published:** 2010-12-11

**Authors:** Krzysztof Brzezinski, Aneta M. Tomkiel, Zenon Łotowski, Jacek Morzycki, Zbigniew Dauter

**Affiliations:** a Synchrotron Radiation Research Section, MCL, National Cancer Institute, Argonne National Laboratory, Argonne, IL 60439, USA; b Institute of Chemistry, University of Białystok, Piłsudskiego 11/4, 15-443 Białystok, Poland

## Abstract

The crystal structure of the title compound, C_90_H_100_N_6_O_24_S_2_·C_4_H_8_O_2_·0.25C_6_H_14_, solved and refined against synchrotron diffraction data, contains two formula units in the asymmetric unit with the all-*trans n*-hexane mol­ecule having half-occupancy and one of the ethyl acetate mol­ecules disordered over two positions. The two symmetry-independent disulfide mol­ecules are assembled by approximate face-to-face and face-to-edge inter­actions between their 4-nitro­benzo­yloxy groups into an inter­twined dimer having a double-helix-type structure. The centrally placed disulfide bridges in the two symmetry-independent mol­ecules exhibit different helicity as shown by the C—S—S—C torsion angles of 71.0 (1) and −92.5 (1)°.

## Related literature

For background to the chemistry of bile acids and their application in asymmetric synthesis, chiral discrimination and recognition, see: Bortolini *et al.* (2010 ▶); Davis (2007 ▶); Mukhopadhyay & Maitra (2004 ▶); Wilson (2007 ▶); Virtanen & Kolehmainen (2004 ▶). For the synthesis of dimeric and oligomeric steroids and their use in supra­molecular chemistry, see: Li & Dias (1997 ▶); McKenna *et al.* (1977 ▶). For properties of natural and synthetic disulfides, see: Fluharty (1974 ▶); Wouters *et al.* (2010 ▶); Houmam (2008 ▶); Szilágyi *et al.* (2001 ▶); Lees & Whitesides (1993 ▶); Mu *et al.* (2004 ▶). For the preparation of bis­(3α,7α,12α-trihy­droxy-5β-cholan-24-yl) disulfide, see: Łotowski & Kulesza (2010 ▶).
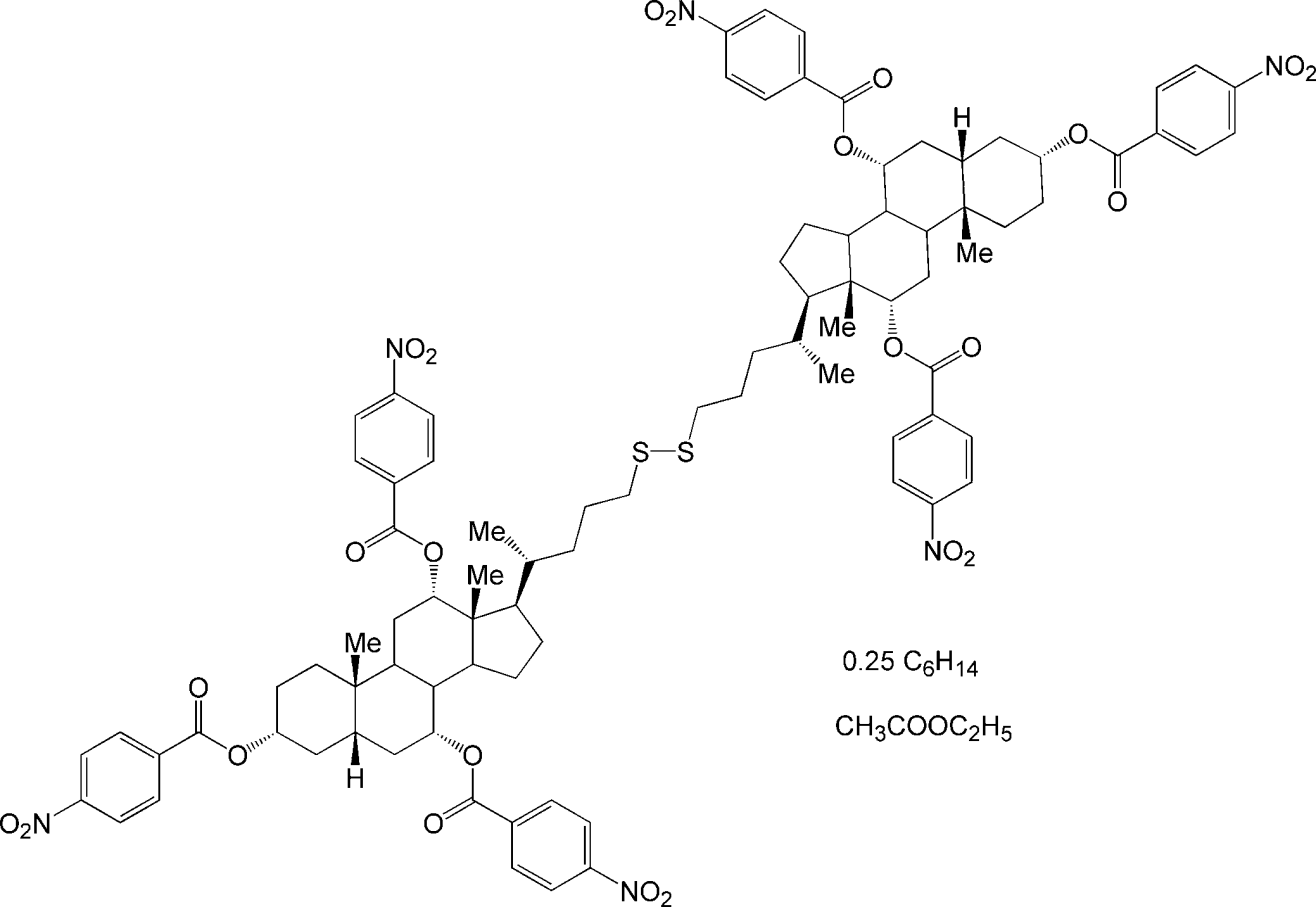


## Experimental

### 

#### Crystal data

C_90_H_100_N_6_O_24_S_2_·C_4_H_8_O_2_·0.25C_6_H_14_
*M* *_r_* = 1823.53
Orthorhombic, 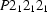

*a* = 22.81 (2) Å
*b* = 26.47 (3) Å
*c* = 30.77 (3) Å
*V* = 18578 (31) Å^3^
*Z* = 8
Synchrotron radiation
λ = 0.59040 Å
μ = 0.09 mm^−1^
*T* = 100 K
0.15 × 0.03 × 0.03 mm

#### Data collection

Mar MAR315 CCD diffractometer
Absorption correction: multi-scan (*SCALEPACK*; Otwinowski *et al.*, 2003 ▶) *T*_min_ = 0.987, *T*_max_ = 0.997
247079 measured reflections
88841 independent reflections
85229 reflections with *I* > 2σ(*I*)
*R*_int_ = 0.043

#### Refinement

*R*[*F* ^2^ > 2σ(*F* ^2^)] = 0.045
*wR*(*F* ^2^) = 0.135
*S* = 1.02
88841 reflections
2324 parameters
38 restraints
H-atom parameters constrained
Δρ_max_ = 1.78 e Å^−3^
Δρ_min_ = −0.81 e Å^−3^
Absolute structure: Flack (1983 ▶), 39133 Friedel pairs
Flack parameter: −0.07 (3)

### 

Data collection: *NECAT APS beamline Software* (unpublished); cell refinement: *HKL-2000* (Otwinowski & Minor, 1997 ▶); data reduction: *HKL-2000*; program(s) used to solve structure: *SHELXD* (Sheldrick, 2008 ▶); program(s) used to refine structure: *SHELXL97* (Sheldrick, 2008 ▶); molecular graphics: *ORTEP-3* (Farrugia, 1997 ▶) and *pyMOL* (DeLano, 2002 ▶); software used to prepare material for publication: *SHELXL97*.

## Supplementary Material

Crystal structure: contains datablocks global, I. DOI: 10.1107/S1600536810050385/gk2316sup1.cif

Structure factors: contains datablocks I. DOI: 10.1107/S1600536810050385/gk2316Isup2.hkl

Additional supplementary materials:  crystallographic information; 3D view; checkCIF report

## Figures and Tables

**Fig. 1.**
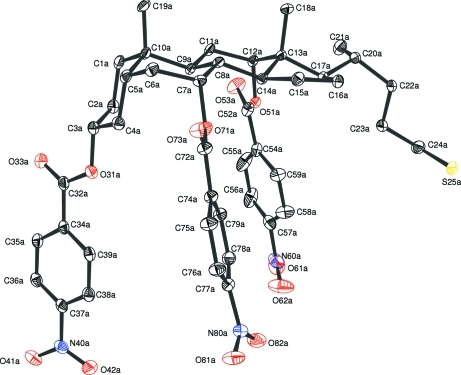
The molecular structure of the title compound. For clarity, only a half of the molecule is shown and hydrogen atoms are omitted. Atoms in the second part of the molecule are marked with a suffix "b" and those in the other independent molecule by suffixes "c" and "d". Displacement ellipsoids are drawn at the 50% probability level.

**Fig. 2.**
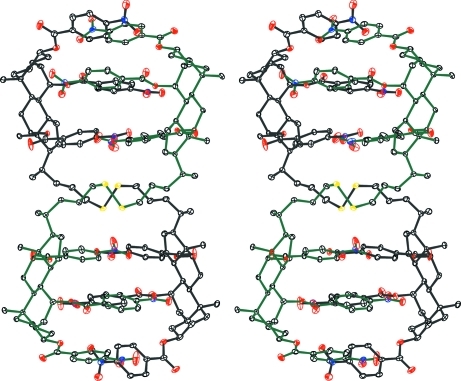
Stereoview representation of a tight dimer present in the asymmetric unit. The green part corresponds to the molecule with atoms marked with a suffix "a" and "b" and the black one with "c" and "d", respectively. Displacement ellipsoids are drawn at the 50% probability level.

**Fig. 3.**
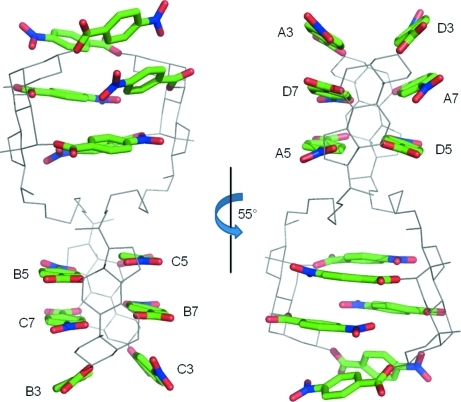
Clusters formed by p-nitrobenzoyloxy moieties within two halves of the dimer. Each aromatic moiety is marked by a letter relating to the parent cholane system and a number showing its position.

**Fig. 4.**
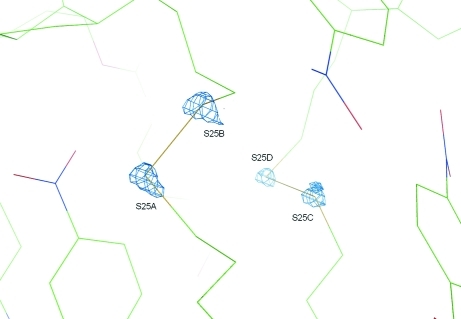
Anomalous difference Fourier synthesis contoured at 0.53 e Å-3 (6.0 σ) level, overlapped on the central fragment of the dimer.

## supplementary crystallographic information

### Comment

Cholic acid derivatives are widely used in supramolecular chemistry. They serve as molecular receptors, enzyme models and transporters across phospholipid membranes. The acyclic dimers of cholic acid derivatives are potential supramolecular hosts for ions and small molecules. The axial hydroxyl groups present in cholic acid may be involved in binding of a guest molecule by formation of multiple hydrogen bonds. The dimers are expected to adopt a suitable conformation for binding of a particular guest molecule and are considered to be molecular clefts. The disulfide linkers allow to extend the available conformational space by increasing distance and flexibility within the connected steroidal units. In addition to that, the disulfide linkers readily undergo a reductive cleavage that may be used for releasing of the guest molecule from the supramolecule. We have recently described (Łotowski & Kulesza, 2010) a straightforward synthesis of bis(3α,7α,12α-trihydroxy-5β-cholan-24-yl) disulfide from cholic acid. The product was found to be crystalline but unfortunately the crystals proved not to be suitable for the X-ray examination.

Therefore the per-4-nitrobenzoyl derivative was prepared and its X-ray structure is reported herein (Fig. 1). The asymmetric unit contains a tight dimer (Fig. 2) consisting of two intertwined molecules of the title compound, two ethyl acetate molecules, where one is disordered and also a disordered and fractionally occupied n-hexane molecule. Each half of the dimer contains a cluster of six 4-nitrobenzoyloxy moieties, mutually interacting in a mixed, π-π-stacking and edge-to-face mode (Fig. 3). The "inner" aromatic rings substituted in cholane positions 5 and 7 display tendency to be parallel, whereas the planes of the outer rings in positions 3 are significantly skewed. In spite of the short wavelength used for data collection at the sychrotron beam line in order to obtain high resolution, providing relatively small value of f"=0.085 of sulfur atoms, the anomalous difference synthesis reveals clear peaks corresponding to positions of all four sulfur atoms, Fig. 4. The anomalous signal of sulfur atoms unequivocally confirmed the chirality of the title compound through close to zero value of the Flack parameter.

### Experimental

Bis(3α,7α,12α-trihydroxy-5β-cholan-24-yl) disulfide (100 mg; 0.12 mmol) was dissolved in dry pyridine and 4-nitrobenzoyl chloride (150 mg; 0.8 mmol) was added. The reaction mixture was stirred for 24 h at room temperature, poured into water, and extracted with dichloromethane. The extract was dried (anhydrous Na_2_SO_4_) and the solvent was evaporated *in vacuo*. The crude product was purified by silica gel column chromatography. Pure bis[3α,7α,12α-tri(4-nitrobenzoyloxy)-5β-cholan-24-yl] disulfide (163 mg; 78%) was eluted with a benzene-ethyl acetate (95:5) mixture.

M.p. 440–443 K (from ethyl acetate - hexane)

MS-ESI, m/*z*: 1735 (MNa+, 100%); HR (MS-ESI), found: 1735.6140; calculated for C_90_H_100_N_6_O_24_S_2_Na: 1735.6128.

1H NMR (400 MHz, CDCl3, selected signals): δ 8.24 (m, 8H, Ar—H); 8.03 (d, *J* = 8.8 Hz, 2H, Ar—H), 7.85 (d, *J* = 8.8 Hz, 2H, Ar—H), 5.46 (m, 1H, 12β-H), 5.37 (m, 1H, 7β-H), 4.78 (m, 1H, 3β-H), 1.09 (s, 3H, 19-H), 0.88 (s, 3H, 18-H), 0.79 (d, *J* = 6.5 Hz, 3H, 21-H).

### Refinement

The n-hexane and one of total two ethyl acetate solvent molecules are disordered. For n-hexane molecule, the occupancy is fixed at 0.5 with isotropic atomic displacement parameters for all atoms. The disordered ethyl acetate molecule is modeled in two alternative conformations which refined isotropically to occupancies of 0.58:0.42. Distance and angle restraints were only applied to the disordered parts of the solvent molecules. The final difference minimum and maximum (-0.81 and 1.78 e Å^-3^) indicate an imperfect modeling of the solvent area region. All hydrogen atoms were constrained to idealized positions with C—H distances fixed at 0.95–1.00 Å and *U*_iso_(H) = 1.5*U*_eq_(C) for methyl hydrogen atoms and 1.2*U*_eq_(C) for others.

### Crystal data

**Table:**

C_90_H_100_N_6_O_24_S_2_·C_4_H_8_O_2_·0.25C_6_H_14_	*F*(000) = 7732
*M**_r_* = 1823.53	*D*_x_ = 1.304 Mg m^−^^3^
Orthorhombic, *P*2_1_2_1_2_1_	Synchrotron radiation, λ = 0.59040 Å
Hall symbol: P 2ac 2ab	Cell parameters from 88841 reflections
*a* = 22.81 (2) Å	θ = 1.0–30.0°
*b* = 26.47 (3) Å	µ = 0.09 mm^−^^1^
*c* = 30.77 (3) Å	*T* = 100 K
*V* = 18578 (31) Å^3^	Needle, yellow
*Z* = 8	0.15 × 0.03 × 0.03 mm

### Data collection

**Table:**

Mar MAR315 CCD diffractometer	88841 independent reflections
Radiation source: NECAT 24ID-C synchrotron beamline APS, USA	85229 reflections with *I* > 2σ(*I*)
Si111 double crystal	*R*_int_ = 0.043
ω scans	θ_max_ = 30.0°, θ_min_ = 1.0°
Absorption correction: multi-scan (*SCALEPACK*; Otwinowski *et al.*, 2003)	*h* = −33→34
*T*_min_ = 0.987, *T*_max_ = 0.997	*k* = −44→44
247079 measured reflections	*l* = −52→52

### Refinement

**Table:**

Refinement on *F*^2^	Secondary atom site location: difference Fourier map
Least-squares matrix: full	Hydrogen site location: inferred from neighbouring sites
*R*[*F*^2^ > 2σ(*F*^2^)] = 0.045	H-atom parameters constrained
*wR*(*F*^2^) = 0.135	*w* = 1/[σ^2^(*F*_o_^2^) + (0.0972*P*)^2^ + 2.7163*P*] where *P* = (*F*_o_^2^ + 2*F*_c_^2^)/3
*S* = 1.02	(Δ/σ)_max_ = 0.023
88841 reflections	Δρ_max_ = 1.78 e Å^−^^3^
2324 parameters	Δρ_min_ = −0.81 e Å^−^^3^
38 restraints	Absolute structure: Flack (1983), 39133 Friedel pairs
Primary atom site location: structure-invariant direct methods	Flack parameter: −0.07 (3)

### Special details

**Table:**

Experimental. The crystal was mounted with vaseline on a pin attached capillary. Upon mounting, the crystal was quenched to 100 K in a nitrogen-gas stream supplied by an Oxford Cryo-Jet. Diffraction data were measured at the station 24-ID—C of the APS synchrotron by rotation method.
Geometry. All esds are estimated using the full covariance matrix. The cell esds are taken into account individually in the estimation of esds in distances, angles and torsion angles; correlations between esds in cell parameters are only used when they are defined by crystal symmetry.
Refinement. Refinement of *F*^2^ against all reflections. The weighted *R*-factor *wR* and goodness of fit *S* are based on *F*^2^, conventional *R*-factors *R* are based on *F*, with *F* set to zero for negative *F*^2^. The threshold expression of *F*^2^ > 2σ(*F*^2^) is used only for calculating *R*-factors *etc*. and is not relevant to the choice of reflections for refinement. *R*-factors based on *F*^2^ are statistically about twice as large as those based on *F*.

### Fractional atomic coordinates and isotropic or equivalent isotropic displacement parameters (Å^2^)

**Table:**

	*x*	*y*	*z*	*U*_iso_*/*U*_eq_	Occ. (<1)
C1A	0.39744 (5)	0.71281 (3)	−0.00177 (3)	0.01787 (15)	
H1A1	0.3955	0.7501	−0.0030	0.021*	
H1A2	0.4162	0.7035	0.0261	0.021*	
C2A	0.43595 (5)	0.69412 (4)	−0.03901 (3)	0.01830 (15)	
H2A1	0.4751	0.7100	−0.0372	0.022*	
H2A2	0.4410	0.6571	−0.0369	0.022*	
C3A	0.40741 (5)	0.70749 (4)	−0.08207 (3)	0.01838 (15)	
H3A1	0.4061	0.7451	−0.0854	0.022*	
C4A	0.34601 (4)	0.68614 (4)	−0.08545 (3)	0.01773 (14)	
H4A1	0.3480	0.6488	−0.0856	0.021*	
H4A2	0.3281	0.6971	−0.1132	0.021*	
C5A	0.30712 (4)	0.70369 (3)	−0.04728 (3)	0.01640 (14)	
H5A1	0.3044	0.7413	−0.0493	0.020*	
C6A	0.24450 (4)	0.68330 (3)	−0.05070 (3)	0.01747 (15)	
H6A1	0.2192	0.7027	−0.0306	0.021*	
H6A2	0.2300	0.6894	−0.0806	0.021*	
C7A	0.23812 (4)	0.62715 (3)	−0.04043 (3)	0.01474 (13)	
H7A1	0.1955	0.6194	−0.0369	0.018*	
C8A	0.27000 (4)	0.61148 (3)	0.00108 (3)	0.01313 (12)	
H8A1	0.2472	0.6258	0.0260	0.016*	
C9A	0.33290 (4)	0.63317 (3)	0.00440 (3)	0.01270 (12)	
H9A1	0.3564	0.6177	−0.0196	0.015*	
C10A	0.33442 (4)	0.69154 (3)	−0.00235 (3)	0.01472 (13)	
C11A	0.36152 (4)	0.61738 (3)	0.04743 (3)	0.01518 (13)	
H11A1	0.4032	0.6278	0.0468	0.018*	
H11A2	0.3423	0.6361	0.0713	0.018*	
C12A	0.35885 (4)	0.56102 (3)	0.05788 (3)	0.01406 (13)	
H12A1	0.3727	0.5556	0.0884	0.017*	
C13A	0.29681 (4)	0.53960 (3)	0.05343 (3)	0.01330 (12)	
C14A	0.27293 (4)	0.55423 (3)	0.00802 (3)	0.01265 (12)	
H14A1	0.3015	0.5408	−0.0138	0.015*	
C15A	0.21700 (4)	0.52233 (4)	0.00354 (3)	0.01780 (14)	
H15A1	0.2083	0.5151	−0.0274	0.021*	
H15A2	0.1830	0.5398	0.0167	0.021*	
C16A	0.23166 (4)	0.47307 (4)	0.02861 (3)	0.01787 (15)	
H16A1	0.2337	0.4441	0.0083	0.021*	
H16A2	0.2011	0.4660	0.0506	0.021*	
C17A	0.29197 (4)	0.48127 (3)	0.05105 (3)	0.01390 (13)	
H17A1	0.3228	0.4693	0.0304	0.017*	
C18A	0.25859 (5)	0.56025 (4)	0.09088 (3)	0.01960 (16)	
H18A1	0.2191	0.5458	0.0889	0.029*	
H18A2	0.2562	0.5971	0.0887	0.029*	
H18A3	0.2762	0.5509	0.1188	0.029*	
C19A	0.30033 (5)	0.71963 (3)	0.03361 (3)	0.01984 (16)	
H19A1	0.2605	0.7058	0.0357	0.030*	
H19A2	0.2983	0.7557	0.0265	0.030*	
H19A3	0.3205	0.7152	0.0615	0.030*	
C20A	0.29841 (5)	0.45018 (3)	0.09309 (3)	0.01825 (15)	
H20A1	0.2631	0.4571	0.1114	0.022*	
C21A	0.35233 (7)	0.46314 (4)	0.12051 (4)	0.0269 (2)	
H21A1	0.3543	0.4404	0.1456	0.040*	
H21A2	0.3495	0.4982	0.1306	0.040*	
H21A3	0.3878	0.4592	0.1028	0.040*	
C22A	0.29841 (5)	0.39315 (3)	0.08260 (3)	0.01973 (16)	
H22A1	0.2993	0.3741	0.1103	0.024*	
H22A2	0.2613	0.3847	0.0676	0.024*	
C23A	0.34964 (5)	0.37558 (4)	0.05427 (4)	0.02173 (17)	
H23A1	0.3431	0.3874	0.0241	0.026*	
H23A2	0.3862	0.3915	0.0650	0.026*	
C24A	0.35779 (6)	0.31859 (4)	0.05386 (4)	0.02347 (18)	
H24A1	0.3600	0.3066	0.0843	0.028*	
H24A2	0.3958	0.3109	0.0399	0.028*	
S25A	0.300740 (15)	0.283047 (9)	0.026104 (9)	0.02349 (5)	
O31A	0.44015 (4)	0.68524 (3)	−0.11847 (3)	0.02075 (13)	
C32A	0.49072 (5)	0.70780 (4)	−0.12887 (3)	0.01941 (16)	
O33A	0.51046 (4)	0.74465 (3)	−0.11063 (3)	0.02588 (16)	
C34A	0.52098 (5)	0.68283 (4)	−0.16633 (3)	0.01775 (15)	
C35A	0.57479 (5)	0.70285 (4)	−0.17909 (4)	0.01972 (16)	
H35A1	0.5903	0.7314	−0.1643	0.024*	
C36A	0.60579 (5)	0.68141 (4)	−0.21317 (4)	0.01990 (16)	
H36A1	0.6424	0.6949	−0.2222	0.024*	
C37A	0.58171 (5)	0.63977 (4)	−0.23368 (3)	0.01877 (15)	
C38A	0.52781 (5)	0.61922 (4)	−0.22223 (3)	0.02057 (16)	
H38A1	0.5124	0.5909	−0.2373	0.025*	
C39A	0.49707 (5)	0.64126 (4)	−0.18815 (3)	0.01935 (15)	
H39A1	0.4600	0.6282	−0.1797	0.023*	
N40A	0.61514 (5)	0.61604 (4)	−0.26915 (3)	0.02271 (16)	
O41A	0.65544 (5)	0.63990 (4)	−0.28530 (3)	0.02936 (18)	
O42A	0.60095 (6)	0.57317 (4)	−0.28005 (4)	0.0332 (2)	
O51A	0.39687 (3)	0.53269 (2)	0.02817 (2)	0.01367 (10)	
C52A	0.45362 (4)	0.52997 (4)	0.03849 (3)	0.01823 (15)	
O53A	0.47661 (5)	0.55300 (4)	0.06785 (3)	0.03032 (19)	
C54A	0.48593 (4)	0.49388 (4)	0.00952 (3)	0.01712 (14)	
C55A	0.54712 (5)	0.49357 (5)	0.01088 (6)	0.0347 (3)	
H55A1	0.5672	0.5153	0.0304	0.042*	
C56A	0.57866 (5)	0.46176 (5)	−0.01615 (7)	0.0356 (3)	
H56A1	0.6203	0.4614	−0.0153	0.043*	
C57A	0.54824 (5)	0.43055 (4)	−0.04440 (4)	0.02311 (19)	
C58A	0.48747 (5)	0.42769 (5)	−0.04453 (4)	0.0256 (2)	
H58A1	0.4677	0.4040	−0.0625	0.031*	
C59A	0.45625 (4)	0.46029 (4)	−0.01779 (4)	0.02076 (17)	
H59A1	0.4146	0.4598	−0.0181	0.025*	
N60A	0.58065 (5)	0.39701 (5)	−0.07410 (4)	0.0310 (2)	
O61A	0.63027 (5)	0.38433 (5)	−0.06306 (5)	0.0400 (3)	
C62A	0.55606 (7)	0.38367 (7)	−0.10746 (4)	0.0521 (4)	
O71A	0.26092 (3)	0.59680 (3)	−0.07658 (2)	0.01485 (11)	
C72A	0.22471 (4)	0.59286 (4)	−0.11061 (3)	0.01674 (14)	
O73A	0.17631 (4)	0.61175 (4)	−0.11254 (3)	0.02551 (16)	
C74A	0.24954 (4)	0.56131 (3)	−0.14633 (3)	0.01567 (13)	
C75A	0.21600 (5)	0.55687 (4)	−0.18417 (3)	0.02032 (16)	
H75A1	0.1805	0.5754	−0.1868	0.024*	
C76A	0.23386 (5)	0.52579 (4)	−0.21788 (3)	0.02086 (16)	
H76A1	0.2116	0.5230	−0.2438	0.025*	
C77A	0.28541 (5)	0.49885 (4)	−0.21235 (3)	0.01782 (14)	
C78A	0.32043 (5)	0.50357 (4)	−0.17582 (3)	0.01856 (15)	
H78A1	0.3561	0.4853	−0.1736	0.022*	
C79A	0.30252 (5)	0.53555 (4)	−0.14238 (3)	0.01750 (14)	
H79A1	0.3262	0.5397	−0.1172	0.021*	
N80A	0.30303 (5)	0.46256 (4)	−0.24602 (3)	0.02323 (16)	
O81A	0.27763 (6)	0.46375 (5)	−0.28104 (3)	0.0347 (2)	
C82A	0.34224 (5)	0.43274 (4)	−0.23709 (3)	0.0322 (2)	
C1B	0.37060 (5)	−0.13479 (3)	−0.01715 (3)	0.01707 (14)	
H1B1	0.3583	−0.1707	−0.0178	0.020*	
H1B2	0.3924	−0.1279	−0.0443	0.020*	
C2B	0.41206 (4)	−0.12708 (4)	0.02124 (3)	0.01714 (14)	
H2B1	0.4460	−0.1502	0.0187	0.021*	
H2B2	0.4269	−0.0919	0.0214	0.021*	
C3B	0.37907 (4)	−0.13777 (3)	0.06293 (3)	0.01586 (14)	
H3B1	0.3674	−0.1742	0.0640	0.019*	
C4B	0.32512 (4)	−0.10457 (4)	0.06690 (3)	0.01518 (13)	
H4B1	0.3375	−0.0688	0.0693	0.018*	
H4B2	0.3037	−0.1136	0.0938	0.018*	
C5B	0.28380 (4)	−0.11034 (3)	0.02762 (3)	0.01455 (13)	
H5B1	0.2700	−0.1462	0.0276	0.017*	
C6B	0.22900 (4)	−0.07705 (4)	0.03234 (3)	0.01630 (14)	
H6B1	0.1982	−0.0901	0.0127	0.020*	
H6B2	0.2142	−0.0801	0.0625	0.020*	
C7B	0.23888 (4)	−0.02131 (3)	0.02228 (3)	0.01399 (12)	
H7B1	0.1999	−0.0042	0.0199	0.017*	
C8B	0.27298 (4)	−0.01212 (3)	−0.01958 (3)	0.01256 (12)	
H8B1	0.2477	−0.0229	−0.0445	0.015*	
C9B	0.32964 (4)	−0.04408 (3)	−0.02073 (3)	0.01162 (11)	
H9B1	0.3531	−0.0345	0.0054	0.014*	
C10B	0.31534 (4)	−0.10143 (3)	−0.01638 (3)	0.01408 (13)	
C11B	0.36733 (4)	−0.03159 (3)	−0.06077 (3)	0.01396 (13)	
H11B1	0.4059	−0.0484	−0.0575	0.017*	
H11B2	0.3481	−0.0460	−0.0868	0.017*	
C12B	0.37763 (4)	0.02487 (3)	−0.06857 (3)	0.01207 (12)	
H12B1	0.3976	0.0295	−0.0972	0.014*	
C13B	0.32039 (4)	0.05529 (3)	−0.06840 (3)	0.01222 (12)	
C14B	0.28750 (4)	0.04358 (3)	−0.02566 (3)	0.01199 (11)	
H14B1	0.3145	0.0531	−0.0014	0.014*	
C15B	0.23693 (4)	0.08172 (4)	−0.02497 (3)	0.01627 (14)	
H15B1	0.2253	0.0899	0.0052	0.020*	
H15B2	0.2024	0.0685	−0.0408	0.020*	
C16B	0.26260 (4)	0.12868 (4)	−0.04821 (3)	0.01638 (14)	
H16B1	0.2652	0.1575	−0.0278	0.020*	
H16B2	0.2373	0.1386	−0.0729	0.020*	
C17B	0.32462 (4)	0.11391 (3)	−0.06463 (3)	0.01296 (12)	
H17B1	0.3530	0.1217	−0.0408	0.016*	
C18B	0.28540 (5)	0.04064 (4)	−0.10918 (3)	0.01800 (15)	
H18B1	0.2483	0.0593	−0.1097	0.027*	
H18B2	0.2773	0.0043	−0.1087	0.027*	
H18B3	0.3083	0.0490	−0.1352	0.027*	
C19B	0.27629 (5)	−0.12037 (4)	−0.05403 (3)	0.02035 (16)	
H19B1	0.2407	−0.0997	−0.0556	0.031*	
H19B2	0.2655	−0.1557	−0.0489	0.031*	
H19B3	0.2979	−0.1177	−0.0815	0.031*	
C20B	0.34334 (5)	0.14467 (3)	−0.10457 (3)	0.01676 (14)	
H20B1	0.3156	0.1363	−0.1287	0.020*	
C21B	0.40512 (5)	0.13110 (4)	−0.12010 (3)	0.02119 (17)	
H21B1	0.4167	0.1540	−0.1436	0.032*	
H21B2	0.4055	0.0962	−0.1308	0.032*	
H21B3	0.4327	0.1344	−0.0959	0.032*	
C22B	0.33858 (5)	0.20211 (4)	−0.09613 (3)	0.01915 (16)	
H22B1	0.3499	0.2203	−0.1230	0.023*	
H22B2	0.2971	0.2104	−0.0899	0.023*	
C23B	0.37639 (5)	0.22153 (4)	−0.05868 (3)	0.02009 (16)	
H23B1	0.3619	0.2068	−0.0311	0.024*	
H23B2	0.4172	0.2098	−0.0630	0.024*	
C24B	0.37637 (6)	0.27880 (4)	−0.05478 (4)	0.02264 (18)	
H24B1	0.3865	0.2933	−0.0835	0.027*	
H24B2	0.4077	0.2887	−0.0342	0.027*	
S25B	0.307740 (15)	0.307124 (10)	−0.036622 (9)	0.02430 (5)	
O31B	0.41481 (3)	−0.12530 (3)	0.10084 (2)	0.01777 (12)	
C32B	0.45929 (5)	−0.15621 (4)	0.11026 (3)	0.01806 (15)	
O33B	0.47074 (4)	−0.19487 (3)	0.09102 (3)	0.02710 (17)	
C34B	0.49385 (4)	−0.13726 (3)	0.14819 (3)	0.01651 (14)	
C35B	0.54872 (5)	−0.15859 (4)	0.15612 (4)	0.02040 (16)	
H35B1	0.5632	−0.1842	0.1374	0.024*	
C36B	0.58225 (5)	−0.14266 (4)	0.19114 (4)	0.02040 (16)	
H36B1	0.6198	−0.1569	0.1966	0.024*	
C37B	0.55952 (5)	−0.10556 (4)	0.21774 (3)	0.01923 (15)	
C38B	0.50507 (5)	−0.08352 (5)	0.21100 (3)	0.02411 (19)	
H38B1	0.4907	−0.0580	0.2300	0.029*	
C39B	0.47211 (5)	−0.09978 (4)	0.17567 (3)	0.02170 (17)	
H39B1	0.4347	−0.0853	0.1702	0.026*	
N40B	0.59430 (5)	−0.08841 (4)	0.25525 (3)	0.02615 (18)	
O41B	0.64302 (4)	−0.10614 (4)	0.26061 (4)	0.03025 (18)	
O42B	0.57177 (8)	−0.05750 (8)	0.27941 (5)	0.0645 (6)	
O51B	0.41519 (3)	0.04507 (2)	−0.03423 (2)	0.01228 (9)	
C52B	0.47303 (4)	0.04119 (4)	−0.04029 (3)	0.01630 (14)	
O53B	0.49586 (4)	0.01923 (5)	−0.07027 (3)	0.0312 (2)	
C54B	0.50710 (4)	0.06847 (3)	−0.00590 (3)	0.01475 (13)	
C55B	0.56801 (4)	0.07195 (4)	−0.01089 (4)	0.02100 (17)	
H55B1	0.5867	0.0558	−0.0347	0.025*	
C56B	0.60111 (4)	0.09910 (4)	0.01918 (4)	0.02185 (17)	
H56B1	0.6424	0.1022	0.0160	0.026*	
C57B	0.57239 (4)	0.12132 (3)	0.05373 (3)	0.01680 (14)	
C58B	0.51250 (4)	0.11703 (4)	0.06026 (3)	0.01714 (14)	
H58B1	0.4944	0.1316	0.0851	0.021*	
C59B	0.47948 (4)	0.09077 (3)	0.02959 (3)	0.01539 (13)	
H59B1	0.4382	0.0881	0.0329	0.018*	
N60B	0.60608 (5)	0.15220 (4)	0.08488 (4)	0.02376 (17)	
O61B	0.65694 (5)	0.16194 (6)	0.07596 (5)	0.0462 (3)	
O62B	0.58076 (6)	0.16708 (5)	0.11759 (3)	0.0351 (2)	
O71B	0.27227 (3)	0.00262 (3)	0.05706 (2)	0.01358 (10)	
C72B	0.24141 (4)	0.02268 (3)	0.08956 (3)	0.01419 (13)	
O73B	0.18862 (3)	0.02004 (4)	0.09321 (3)	0.02194 (14)	
C74B	0.27988 (4)	0.04947 (3)	0.12137 (3)	0.01371 (13)	
C75B	0.25399 (4)	0.06684 (4)	0.15946 (3)	0.01861 (15)	
H75B1	0.2134	0.0612	0.1643	0.022*	
C76B	0.28687 (5)	0.09226 (4)	0.19040 (3)	0.01923 (15)	
H76B1	0.2695	0.1042	0.2165	0.023*	
C77B	0.34589 (4)	0.09975 (3)	0.18201 (3)	0.01640 (14)	
C78B	0.37296 (4)	0.08317 (4)	0.14424 (3)	0.01675 (14)	
H78B1	0.4135	0.0893	0.1394	0.020*	
C79B	0.33950 (4)	0.05738 (3)	0.11357 (3)	0.01521 (13)	
H79B1	0.3571	0.0453	0.0876	0.018*	
N80B	0.38156 (5)	0.12667 (4)	0.21443 (3)	0.02099 (15)	
O81B	0.35585 (5)	0.14831 (4)	0.24411 (3)	0.03005 (18)	
O82B	0.43501 (5)	0.12633 (4)	0.20989 (3)	0.02926 (17)	
C1C	0.49411 (5)	0.16135 (5)	0.35029 (3)	0.02082 (17)	
H1C1	0.4989	0.1505	0.3809	0.025*	
H1C2	0.4753	0.1950	0.3506	0.025*	
C2C	0.45320 (5)	0.12420 (5)	0.32743 (3)	0.02236 (18)	
H2C1	0.4155	0.1223	0.3432	0.027*	
H2C2	0.4452	0.1360	0.2975	0.027*	
C3C	0.48170 (5)	0.07242 (5)	0.32611 (3)	0.02146 (17)	
H3C1	0.4850	0.0581	0.3561	0.026*	
C4C	0.54113 (5)	0.07428 (4)	0.30446 (4)	0.01992 (16)	
H4C1	0.5362	0.0836	0.2735	0.024*	
H4C2	0.5592	0.0403	0.3056	0.024*	
C5C	0.58223 (4)	0.11257 (4)	0.32649 (3)	0.01805 (15)	
H5C1	0.5882	0.1006	0.3570	0.022*	
C6C	0.64277 (4)	0.11281 (4)	0.30484 (4)	0.01968 (16)	
H6C1	0.6713	0.1284	0.3251	0.024*	
H6C2	0.6552	0.0774	0.2999	0.024*	
C7C	0.64526 (4)	0.14105 (4)	0.26161 (3)	0.01698 (14)	
H7C1	0.6871	0.1450	0.2526	0.020*	
C8C	0.61652 (4)	0.19313 (3)	0.26394 (3)	0.01447 (13)	
H8C1	0.6407	0.2145	0.2839	0.017*	
C9C	0.55410 (4)	0.19027 (3)	0.28305 (3)	0.01387 (12)	
H9C1	0.5308	0.1672	0.2640	0.017*	
C10C	0.55538 (4)	0.16641 (4)	0.32947 (3)	0.01623 (14)	
C11C	0.52374 (4)	0.24225 (4)	0.28228 (3)	0.01686 (14)	
H11C1	0.4817	0.2373	0.2890	0.020*	
H11C2	0.5407	0.2632	0.3058	0.020*	
C12C	0.52838 (4)	0.27174 (3)	0.23942 (3)	0.01571 (14)	
H12C1	0.5141	0.3070	0.2443	0.019*	
C13C	0.59123 (5)	0.27383 (3)	0.22208 (3)	0.01635 (14)	
C14C	0.61512 (4)	0.21923 (3)	0.22001 (3)	0.01535 (13)	
H14C1	0.5877	0.1995	0.2012	0.018*	
C15C	0.67286 (5)	0.22437 (4)	0.19491 (4)	0.02140 (17)	
H15C1	0.6825	0.1926	0.1794	0.026*	
H15C2	0.7056	0.2330	0.2147	0.026*	
C16C	0.66106 (6)	0.26767 (4)	0.16247 (4)	0.02384 (19)	
H16C1	0.6615	0.2547	0.1323	0.029*	
H16C2	0.6915	0.2941	0.1652	0.029*	
C17C	0.59973 (5)	0.28965 (4)	0.17374 (3)	0.01931 (16)	
H17C1	0.5703	0.2705	0.1562	0.023*	
C18C	0.62741 (5)	0.30835 (4)	0.25229 (3)	0.02217 (17)	
H18C1	0.6682	0.3093	0.2423	0.033*	
H18C2	0.6260	0.2951	0.2820	0.033*	
H18C3	0.6110	0.3426	0.2518	0.033*	
C19C	0.59163 (5)	0.19900 (4)	0.36106 (3)	0.02176 (17)	
H19C1	0.6307	0.2049	0.3488	0.033*	
H19C2	0.5954	0.1814	0.3889	0.033*	
H19C3	0.5719	0.2315	0.3656	0.033*	
C20C	0.59386 (7)	0.34568 (4)	0.16110 (4)	0.0268 (2)	
H20C1	0.6248	0.3646	0.1774	0.032*	
C21C	0.53507 (8)	0.36914 (5)	0.17305 (4)	0.0340 (3)	
H21C1	0.5336	0.4040	0.1624	0.051*	
H21C2	0.5304	0.3689	0.2047	0.051*	
H21C3	0.5033	0.3496	0.1597	0.051*	
C22C	0.60632 (7)	0.35301 (5)	0.11223 (4)	0.0288 (2)	
H22C1	0.6051	0.3896	0.1056	0.035*	
H22C2	0.6465	0.3409	0.1060	0.035*	
C23C	0.56360 (6)	0.32576 (5)	0.08205 (3)	0.0254 (2)	
H23C1	0.5649	0.2890	0.0880	0.030*	
H23C2	0.5232	0.3377	0.0879	0.030*	
C24C	0.57837 (7)	0.33503 (5)	0.03464 (4)	0.0283 (2)	
H24C1	0.6169	0.3196	0.0282	0.034*	
H24C2	0.5819	0.3719	0.0298	0.034*	
S25C	0.524406 (15)	0.309701 (11)	−0.002788 (9)	0.02524 (5)	
O31C	0.44563 (4)	0.03938 (4)	0.29843 (3)	0.02471 (16)	
C32C	0.40619 (5)	0.00941 (4)	0.31716 (3)	0.01905 (15)	
O33C	0.39949 (5)	0.00356 (5)	0.35569 (3)	0.0316 (2)	
C34C	0.36846 (4)	−0.01583 (4)	0.28380 (3)	0.01612 (14)	
C35C	0.32132 (5)	−0.04498 (4)	0.29822 (3)	0.01975 (16)	
H35C1	0.3156	−0.0501	0.3285	0.024*	
C36C	0.28269 (5)	−0.06662 (4)	0.26858 (3)	0.01972 (16)	
H36C1	0.2505	−0.0866	0.2781	0.024*	
C37C	0.29258 (4)	−0.05818 (3)	0.22473 (3)	0.01610 (14)	
C38C	0.33937 (4)	−0.02977 (4)	0.20926 (3)	0.01694 (14)	
H38C1	0.3451	−0.0250	0.1790	0.020*	
C39C	0.37750 (4)	−0.00856 (4)	0.23934 (3)	0.01670 (14)	
H39C1	0.4099	0.0110	0.2296	0.020*	
N40C	0.25147 (4)	−0.07931 (4)	0.19287 (3)	0.01984 (14)	
O41C	0.21508 (5)	−0.10988 (4)	0.20549 (4)	0.0336 (2)	
O42C	0.25495 (4)	−0.06502 (4)	0.15501 (3)	0.02331 (14)	
O51C	0.49201 (3)	0.24781 (3)	0.20600 (2)	0.01517 (11)	
C52C	0.43567 (5)	0.26099 (4)	0.20518 (3)	0.01951 (16)	
O53C	0.41191 (5)	0.28808 (5)	0.23137 (4)	0.0374 (3)	
C54C	0.40354 (4)	0.23837 (3)	0.16746 (3)	0.01545 (14)	
C55C	0.34247 (5)	0.23473 (4)	0.17046 (3)	0.01811 (15)	
H55C1	0.3228	0.2475	0.1954	0.022*	
C56C	0.31059 (4)	0.21248 (4)	0.13704 (3)	0.01646 (14)	
H56C1	0.2692	0.2093	0.1389	0.020*	
C57C	0.34079 (4)	0.19497 (3)	0.10093 (3)	0.01430 (13)	
C58C	0.40091 (4)	0.20040 (4)	0.09624 (3)	0.01563 (13)	
H58C1	0.4200	0.1897	0.0704	0.019*	
C59C	0.43277 (4)	0.22182 (4)	0.13022 (3)	0.01640 (14)	
H59C1	0.4741	0.2251	0.1281	0.020*	
N60C	0.30840 (4)	0.16705 (3)	0.06742 (3)	0.01738 (13)	
O61C	0.25502 (4)	0.16641 (4)	0.06941 (3)	0.02869 (17)	
O62C	0.33743 (4)	0.14449 (3)	0.03987 (3)	0.02280 (14)	
O71C	0.61405 (3)	0.11156 (3)	0.22857 (3)	0.01714 (12)	
C72C	0.64591 (4)	0.08633 (3)	0.19945 (3)	0.01622 (14)	
O73C	0.69885 (4)	0.08693 (4)	0.19709 (4)	0.02586 (17)	
C74C	0.60805 (4)	0.05629 (3)	0.16922 (3)	0.01564 (14)	
C75C	0.63491 (5)	0.03158 (4)	0.13459 (4)	0.02183 (17)	
H75C1	0.6762	0.0337	0.1311	0.026*	
C76C	0.60192 (5)	0.00389 (4)	0.10508 (4)	0.02350 (18)	
H76C1	0.6200	−0.0132	0.0814	0.028*	
C77C	0.54185 (4)	0.00200 (3)	0.11127 (3)	0.01961 (16)	
C78C	0.51407 (5)	0.02506 (4)	0.14588 (4)	0.02223 (17)	
H78C1	0.4729	0.0220	0.1496	0.027*	
C79C	0.54757 (5)	0.05277 (4)	0.17506 (4)	0.02114 (17)	
H79C1	0.5293	0.0693	0.1989	0.025*	
N80C	0.50516 (4)	−0.02383 (4)	0.07846 (3)	0.02719 (19)	
O81C	0.52969 (6)	−0.05046 (6)	0.05168 (5)	0.0523 (4)	
O82C	0.45231 (4)	−0.01646 (4)	0.07950 (4)	0.03153 (19)	
C1D	0.45527 (5)	0.39465 (4)	−0.35541 (3)	0.01925 (15)	
H1D1	0.4566	0.4036	−0.3866	0.023*	
H1D2	0.4374	0.3607	−0.3530	0.023*	
C2D	0.41579 (5)	0.43251 (4)	−0.33204 (3)	0.01935 (15)	
H2D1	0.3764	0.4328	−0.3456	0.023*	
H2D2	0.4114	0.4228	−0.3011	0.023*	
C3D	0.44334 (5)	0.48434 (4)	−0.33530 (3)	0.01961 (16)	
H3D1	0.4454	0.4952	−0.3664	0.024*	
C4D	0.50401 (5)	0.48498 (4)	−0.31525 (3)	0.01893 (15)	
H4D1	0.5011	0.4769	−0.2839	0.023*	
H4D2	0.5211	0.5192	−0.3181	0.023*	
C5D	0.54438 (4)	0.44642 (4)	−0.33765 (3)	0.01747 (15)	
H5D1	0.5476	0.4571	−0.3687	0.021*	
C6D	0.60671 (4)	0.44841 (4)	−0.31877 (4)	0.01890 (16)	
H6D1	0.6340	0.4326	−0.3398	0.023*	
H6D2	0.6184	0.4842	−0.3155	0.023*	
C7D	0.61345 (4)	0.42214 (3)	−0.27504 (3)	0.01575 (14)	
H7D1	0.6561	0.4197	−0.2677	0.019*	
C8D	0.58681 (4)	0.36924 (3)	−0.27496 (3)	0.01317 (12)	
H8D1	0.6103	0.3481	−0.2955	0.016*	
C9D	0.52286 (4)	0.36914 (3)	−0.29131 (3)	0.01258 (12)	
H9D1	0.4998	0.3917	−0.2716	0.015*	
C10D	0.51887 (4)	0.39198 (4)	−0.33794 (3)	0.01580 (14)	
C11D	0.49595 (4)	0.31593 (3)	−0.28813 (3)	0.01366 (12)	
H11D1	0.4533	0.3185	−0.2938	0.016*	
H11D2	0.5131	0.2947	−0.3114	0.016*	
C12D	0.50486 (4)	0.28897 (3)	−0.24441 (3)	0.01180 (12)	
H12D1	0.4905	0.2534	−0.2466	0.014*	
C13D	0.56943 (4)	0.28907 (3)	−0.23038 (3)	0.01164 (11)	
C14D	0.59046 (4)	0.34446 (3)	−0.23039 (3)	0.01290 (12)	
H14D1	0.5638	0.3637	−0.2106	0.015*	
C15D	0.65050 (4)	0.34237 (4)	−0.20854 (4)	0.01841 (15)	
H15D1	0.6597	0.3748	−0.1941	0.022*	
H15D2	0.6816	0.3347	−0.2300	0.022*	
C16D	0.64469 (4)	0.29934 (4)	−0.17492 (3)	0.01802 (15)	
H16D1	0.6469	0.3131	−0.1450	0.022*	
H16D2	0.6767	0.2744	−0.1788	0.022*	
C17D	0.58412 (4)	0.27382 (3)	−0.18262 (3)	0.01365 (12)	
H17D1	0.5550	0.2907	−0.1632	0.016*	
C18D	0.60418 (4)	0.25574 (3)	−0.26228 (3)	0.01608 (14)	
H18D1	0.6451	0.2536	−0.2528	0.024*	
H18D2	0.6026	0.2706	−0.2914	0.024*	
H18D3	0.5870	0.2218	−0.2630	0.024*	
C19D	0.55335 (5)	0.36006 (4)	−0.37111 (3)	0.02124 (17)	
H19D1	0.5945	0.3579	−0.3621	0.032*	
H19D2	0.5510	0.3759	−0.3998	0.032*	
H19D3	0.5365	0.3260	−0.3725	0.032*	
C20D	0.58571 (5)	0.21732 (3)	−0.17068 (3)	0.01755 (14)	
H20D1	0.6157	0.2011	−0.1898	0.021*	
C21D	0.52756 (5)	0.19033 (4)	−0.17918 (4)	0.02278 (18)	
H21D1	0.5188	0.1912	−0.2103	0.034*	
H21D2	0.4961	0.2074	−0.1631	0.034*	
H21D3	0.5305	0.1551	−0.1695	0.034*	
C22D	0.60558 (5)	0.20903 (4)	−0.12333 (4)	0.02262 (18)	
H22D1	0.6114	0.1724	−0.1187	0.027*	
H22D2	0.6440	0.2257	−0.1193	0.027*	
C23D	0.56340 (6)	0.22875 (5)	−0.08842 (3)	0.0254 (2)	
H23D1	0.5560	0.2652	−0.0933	0.030*	
H23D2	0.5255	0.2107	−0.0907	0.030*	
C24D	0.58884 (8)	0.22104 (6)	−0.04325 (4)	0.0348 (3)	
H24D1	0.6232	0.2435	−0.0397	0.042*	
H24D2	0.6027	0.1857	−0.0407	0.042*	
S25D	0.53681 (2)	0.233816 (12)	0.000664 (9)	0.03257 (7)	
O31D	0.40928 (4)	0.52090 (3)	−0.31008 (3)	0.02126 (14)	
C32D	0.37069 (5)	0.54936 (4)	−0.33155 (3)	0.01948 (16)	
O33D	0.35968 (5)	0.54640 (5)	−0.36981 (3)	0.0354 (2)	
C34D	0.34197 (4)	0.58646 (3)	−0.30167 (3)	0.01541 (13)	
C35D	0.29490 (5)	0.61430 (4)	−0.31766 (4)	0.02030 (17)	
H35D1	0.2825	0.6099	−0.3469	0.024*	
C36D	0.26593 (5)	0.64853 (4)	−0.29103 (3)	0.01971 (16)	
H36D1	0.2338	0.6678	−0.3016	0.024*	
C37D	0.28522 (4)	0.65367 (3)	−0.24864 (3)	0.01539 (13)	
C38D	0.33249 (4)	0.62698 (4)	−0.23187 (3)	0.01748 (14)	
H38D1	0.3449	0.6318	−0.2027	0.021*	
C39D	0.36102 (4)	0.59317 (4)	−0.25892 (3)	0.01705 (14)	
H39D1	0.3936	0.5745	−0.2484	0.020*	
N40D	0.25423 (4)	0.68876 (4)	−0.21980 (3)	0.02157 (15)	
O41D	0.22568 (6)	0.72274 (5)	−0.23644 (4)	0.0404 (3)	
O42D	0.25928 (6)	0.68257 (4)	−0.18048 (3)	0.0327 (2)	
O51D	0.47122 (3)	0.31589 (3)	−0.21110 (2)	0.01244 (10)	
C52D	0.41452 (4)	0.30396 (4)	−0.20728 (3)	0.01687 (14)	
O53D	0.38990 (4)	0.27157 (5)	−0.22809 (4)	0.0372 (3)	
C54D	0.38403 (4)	0.33617 (3)	−0.17451 (3)	0.01321 (12)	
C55D	0.32457 (4)	0.32726 (4)	−0.16699 (3)	0.01741 (15)	
H55D1	0.3051	0.3011	−0.1824	0.021*	
C56D	0.29363 (4)	0.35642 (4)	−0.13718 (3)	0.01678 (14)	
H56D1	0.2531	0.3507	−0.1319	0.020*	
C57D	0.32386 (4)	0.39406 (3)	−0.11555 (3)	0.01373 (12)	
C58D	0.38287 (4)	0.40406 (3)	−0.12262 (3)	0.01414 (13)	
H58D1	0.4020	0.4306	−0.1074	0.017*	
C59D	0.41343 (4)	0.37460 (3)	−0.15232 (3)	0.01381 (12)	
H59D1	0.4539	0.3805	−0.1575	0.017*	
N60D	0.29270 (4)	0.42549 (3)	−0.08349 (3)	0.01683 (13)	
O61D	0.23974 (4)	0.42136 (4)	−0.08080 (3)	0.02666 (17)	
O62D	0.32202 (4)	0.45473 (3)	−0.06130 (3)	0.02434 (15)	
O71D	0.58340 (3)	0.45069 (3)	−0.24092 (3)	0.01595 (11)	
C72D	0.61483 (4)	0.48316 (3)	−0.21741 (3)	0.01478 (13)	
O73D	0.66633 (4)	0.49258 (3)	−0.22305 (3)	0.02200 (14)	
C74D	0.57885 (4)	0.50582 (3)	−0.18180 (3)	0.01506 (13)	
C75D	0.60663 (5)	0.53748 (4)	−0.15212 (4)	0.02044 (16)	
H75D1	0.6470	0.5452	−0.1556	0.025*	
C76D	0.57536 (5)	0.55795 (4)	−0.11727 (4)	0.02264 (18)	
H76D1	0.5941	0.5789	−0.0964	0.027*	
C77D	0.51636 (5)	0.54673 (4)	−0.11410 (3)	0.01876 (15)	
C78D	0.48735 (5)	0.51585 (4)	−0.14344 (4)	0.02025 (16)	
H78D1	0.4466	0.5092	−0.1404	0.024*	
C79D	0.51917 (5)	0.49491 (4)	−0.17744 (3)	0.01849 (15)	
H79D1	0.5004	0.4732	−0.1977	0.022*	
N80D	0.48281 (5)	0.56786 (4)	−0.07771 (3)	0.02325 (17)	
O81D	0.50988 (6)	0.58863 (4)	−0.04841 (3)	0.0332 (2)	
O82D	0.42939 (5)	0.56322 (4)	−0.07798 (4)	0.03127 (19)	
C101	0.27935 (6)	0.07993 (5)	0.32421 (5)	0.0267 (2)	
H101A	0.2475	0.0619	0.3095	0.040*	
H101B	0.2895	0.0622	0.3512	0.040*	
H101C	0.3138	0.0812	0.3052	0.040*	
C102	0.25987 (5)	0.13281 (4)	0.33470 (4)	0.02081 (16)	
O103	0.21117 (5)	0.14894 (5)	0.32892 (5)	0.0434 (3)	
O104	0.30271 (4)	0.15928 (3)	0.35256 (3)	0.02409 (15)	
C105	0.29103 (5)	0.21166 (4)	0.36249 (5)	0.0290 (2)	
H105A	0.2767	0.2149	0.3927	0.035*	
H105B	0.2606	0.2251	0.3427	0.035*	
C106	0.34692 (5)	0.24055 (5)	0.35691 (6)	0.0309 (3)	
H106A	0.3772	0.2262	0.3759	0.046*	
H106B	0.3404	0.2760	0.3646	0.046*	
H106C	0.3598	0.2383	0.3266	0.046*	
C201	0.1947 (2)	−0.17874 (17)	0.1043 (2)	0.091 (2)*	0.583 (4)
H201A	0.1542	−0.1898	0.0993	0.136*	0.583 (4)
H201B	0.2152	−0.1763	0.0764	0.136*	0.583 (4)
H201C	0.1945	−0.1456	0.1185	0.136*	0.583 (4)
C202	0.22594 (12)	−0.21715 (9)	0.13349 (9)	0.0479 (7)*	0.583 (4)
O203	0.27691 (14)	−0.21049 (17)	0.14505 (17)	0.1100 (18)*	0.583 (4)
O204	0.19616 (11)	−0.25912 (9)	0.14592 (8)	0.0519 (7)*	0.583 (4)
C205	0.2247 (2)	−0.28464 (14)	0.18109 (14)	0.0725 (13)*	0.583 (4)
H205A	0.2214	−0.2645	0.2081	0.087*	0.583 (4)
H205B	0.2668	−0.2893	0.1744	0.087*	0.583 (4)
C206	0.19492 (16)	−0.33608 (11)	0.18706 (13)	0.0460 (7)*	0.583 (4)
H206A	0.2138	−0.3543	0.2110	0.069*	0.583 (4)
H206B	0.1986	−0.3558	0.1602	0.069*	0.583 (4)
H206C	0.1533	−0.3310	0.1938	0.069*	0.583 (4)
C211	0.19547 (15)	−0.32793 (12)	0.19968 (10)	0.0304 (6)*	0.417 (4)
H211A	0.1781	−0.3064	0.2221	0.046*	0.417 (4)
H211B	0.2244	−0.3505	0.2130	0.046*	0.417 (4)
H211C	0.1647	−0.3481	0.1858	0.046*	0.417 (4)
C212	0.22553 (10)	−0.29489 (8)	0.16550 (7)	0.0283 (6)*	0.417 (4)
O213	0.25011 (14)	−0.31324 (12)	0.13371 (9)	0.0459 (8)*	0.417 (4)
O214	0.2247 (2)	−0.24389 (8)	0.17137 (12)	0.0787 (16)*	0.417 (4)
C215	0.24943 (19)	−0.21952 (18)	0.1340 (2)	0.181 (8)*	0.417 (4)
H215A	0.2709	−0.2446	0.1161	0.217*	0.417 (4)
H215B	0.2776	−0.1932	0.1433	0.217*	0.417 (4)
C216	0.2004 (2)	−0.1955 (2)	0.10705 (15)	0.0490 (11)*	0.417 (4)
H216A	0.2172	−0.1794	0.0812	0.074*	0.417 (4)
H216B	0.1801	−0.1700	0.1246	0.074*	0.417 (4)
H216C	0.1725	−0.2217	0.0981	0.074*	0.417 (4)
C301	0.0839 (3)	−0.35545 (14)	0.02640 (18)	0.0813 (18)*	0.50
H301A	0.0735	−0.3884	0.0140	0.122*	0.50
H301B	0.0560	−0.3468	0.0494	0.122*	0.50
H301C	0.1236	−0.3570	0.0385	0.122*	0.50
C302	0.0817 (3)	−0.31514 (11)	−0.00918 (13)	0.0606 (11)*	0.50
H302A	0.0416	−0.3136	−0.0214	0.073*	0.50
H302B	0.1089	−0.3245	−0.0329	0.073*	0.50
C303	0.0986 (2)	−0.26322 (11)	0.00876 (11)	0.0584 (11)*	0.50
H303A	0.0749	−0.2557	0.0349	0.070*	0.50
H303B	0.1404	−0.2634	0.0173	0.070*	0.50
C304	0.0882 (2)	−0.22238 (11)	−0.02552 (13)	0.0632 (12)*	0.50
H304A	0.1071	−0.2326	−0.0531	0.076*	0.50
H304B	0.0456	−0.2191	−0.0309	0.076*	0.50
C305	0.1127 (3)	−0.17133 (13)	−0.01122 (16)	0.0795 (17)*	0.50
H305A	0.1558	−0.1739	−0.0076	0.095*	0.50
H305B	0.0956	−0.1618	0.0172	0.095*	0.50
C306	0.0985 (2)	−0.13067 (12)	−0.04494 (14)	0.0532 (9)*	0.50
H306A	0.1148	−0.0982	−0.0354	0.080*	0.50
H306B	0.0559	−0.1276	−0.0480	0.080*	0.50
H306C	0.1157	−0.1401	−0.0730	0.080*	0.50

### Atomic displacement parameters (Å^2^)

**Table:**

	*U*^11^	*U*^22^	*U*^33^	*U*^12^	*U*^13^	*U*^23^
C1A	0.0237 (4)	0.0110 (3)	0.0189 (3)	−0.0017 (3)	−0.0031 (3)	0.0002 (3)
C2A	0.0188 (4)	0.0157 (3)	0.0204 (4)	−0.0006 (3)	−0.0018 (3)	0.0026 (3)
C3A	0.0222 (4)	0.0150 (3)	0.0179 (3)	0.0008 (3)	0.0013 (3)	0.0033 (3)
C4A	0.0192 (4)	0.0183 (3)	0.0157 (3)	0.0021 (3)	−0.0013 (3)	0.0028 (3)
C5A	0.0203 (4)	0.0115 (3)	0.0174 (3)	0.0038 (3)	−0.0021 (3)	0.0019 (2)
C6A	0.0184 (4)	0.0146 (3)	0.0194 (4)	0.0060 (3)	−0.0032 (3)	0.0011 (3)
C7A	0.0150 (3)	0.0152 (3)	0.0140 (3)	0.0043 (2)	−0.0023 (2)	−0.0013 (2)
C8A	0.0151 (3)	0.0114 (3)	0.0128 (3)	0.0017 (2)	−0.0016 (2)	−0.0011 (2)
C9A	0.0166 (3)	0.0090 (3)	0.0125 (3)	0.0007 (2)	−0.0029 (2)	−0.0003 (2)
C10A	0.0196 (4)	0.0091 (3)	0.0154 (3)	0.0012 (2)	−0.0013 (3)	0.0001 (2)
C11A	0.0218 (4)	0.0099 (3)	0.0138 (3)	−0.0007 (2)	−0.0055 (3)	−0.0009 (2)
C12A	0.0204 (4)	0.0107 (3)	0.0111 (3)	−0.0006 (2)	−0.0044 (2)	−0.0005 (2)
C13A	0.0184 (4)	0.0105 (3)	0.0110 (3)	−0.0006 (2)	−0.0004 (2)	−0.0013 (2)
C14A	0.0144 (3)	0.0118 (3)	0.0118 (3)	−0.0004 (2)	−0.0020 (2)	−0.0010 (2)
C15A	0.0160 (4)	0.0167 (3)	0.0207 (4)	−0.0018 (3)	−0.0020 (3)	−0.0016 (3)
C16A	0.0180 (4)	0.0151 (3)	0.0206 (4)	−0.0045 (3)	0.0016 (3)	−0.0014 (3)
C17A	0.0190 (4)	0.0105 (3)	0.0122 (3)	−0.0015 (2)	0.0012 (2)	−0.0007 (2)
C18A	0.0284 (5)	0.0162 (3)	0.0142 (3)	0.0006 (3)	0.0042 (3)	−0.0033 (3)
C19A	0.0274 (5)	0.0115 (3)	0.0206 (4)	0.0031 (3)	0.0000 (3)	−0.0040 (3)
C20A	0.0287 (5)	0.0129 (3)	0.0132 (3)	0.0002 (3)	0.0037 (3)	0.0016 (2)
C21A	0.0458 (7)	0.0179 (4)	0.0168 (4)	−0.0011 (4)	−0.0078 (4)	0.0030 (3)
C22A	0.0279 (5)	0.0116 (3)	0.0196 (4)	−0.0008 (3)	0.0084 (3)	0.0021 (3)
C23A	0.0284 (5)	0.0146 (3)	0.0222 (4)	−0.0005 (3)	0.0085 (3)	0.0008 (3)
C24A	0.0318 (5)	0.0166 (4)	0.0220 (4)	0.0048 (3)	0.0025 (4)	0.0007 (3)
S25A	0.03634 (14)	0.01226 (8)	0.02187 (10)	0.00008 (8)	0.00516 (10)	0.00221 (7)
O31A	0.0219 (3)	0.0198 (3)	0.0205 (3)	−0.0011 (2)	0.0032 (3)	0.0023 (2)
C32A	0.0227 (4)	0.0155 (3)	0.0200 (4)	0.0007 (3)	0.0003 (3)	0.0053 (3)
O33A	0.0307 (4)	0.0194 (3)	0.0275 (4)	−0.0045 (3)	0.0048 (3)	0.0004 (3)
C34A	0.0187 (4)	0.0155 (3)	0.0191 (3)	0.0016 (3)	−0.0007 (3)	0.0061 (3)
C35A	0.0202 (4)	0.0162 (3)	0.0227 (4)	0.0006 (3)	−0.0003 (3)	0.0044 (3)
C36A	0.0179 (4)	0.0198 (4)	0.0220 (4)	0.0027 (3)	0.0005 (3)	0.0064 (3)
C37A	0.0208 (4)	0.0190 (3)	0.0165 (3)	0.0047 (3)	−0.0019 (3)	0.0066 (3)
C38A	0.0227 (4)	0.0199 (4)	0.0192 (4)	0.0006 (3)	−0.0033 (3)	0.0043 (3)
C39A	0.0203 (4)	0.0182 (3)	0.0195 (4)	−0.0005 (3)	−0.0018 (3)	0.0055 (3)
N40A	0.0259 (4)	0.0261 (4)	0.0161 (3)	0.0071 (3)	−0.0016 (3)	0.0057 (3)
O41A	0.0274 (4)	0.0415 (5)	0.0192 (3)	0.0023 (4)	0.0015 (3)	0.0053 (3)
O42A	0.0479 (6)	0.0248 (4)	0.0269 (4)	0.0061 (4)	0.0039 (4)	−0.0008 (3)
O51A	0.0147 (3)	0.0126 (2)	0.0138 (2)	0.00049 (19)	−0.0045 (2)	−0.00094 (18)
C52A	0.0167 (4)	0.0164 (3)	0.0216 (4)	−0.0004 (3)	−0.0083 (3)	0.0000 (3)
O53A	0.0261 (4)	0.0322 (4)	0.0327 (4)	0.0010 (3)	−0.0168 (3)	−0.0111 (3)
C54A	0.0128 (3)	0.0153 (3)	0.0232 (4)	−0.0006 (3)	−0.0046 (3)	0.0025 (3)
C55A	0.0137 (4)	0.0230 (5)	0.0675 (10)	−0.0021 (3)	−0.0056 (5)	−0.0107 (5)
C56A	0.0123 (4)	0.0230 (5)	0.0715 (10)	−0.0029 (3)	0.0045 (5)	−0.0025 (5)
C57A	0.0196 (4)	0.0232 (4)	0.0266 (4)	0.0071 (3)	0.0046 (3)	0.0094 (3)
C58A	0.0185 (4)	0.0349 (5)	0.0233 (4)	0.0114 (4)	−0.0071 (3)	−0.0069 (4)
C59A	0.0138 (4)	0.0264 (4)	0.0221 (4)	0.0039 (3)	−0.0057 (3)	−0.0047 (3)
N60A	0.0283 (5)	0.0321 (5)	0.0324 (5)	0.0135 (4)	0.0104 (4)	0.0100 (4)
O61A	0.0202 (4)	0.0360 (5)	0.0638 (8)	0.0107 (4)	0.0118 (5)	0.0053 (5)
C62A	0.0593 (8)	0.0699 (10)	0.0273 (5)	0.0380 (8)	0.0013 (5)	−0.0047 (5)
O71A	0.0157 (3)	0.0160 (2)	0.0128 (2)	0.0039 (2)	−0.0043 (2)	−0.00146 (19)
C72A	0.0170 (4)	0.0187 (3)	0.0146 (3)	0.0028 (3)	−0.0051 (3)	−0.0019 (3)
O73A	0.0186 (3)	0.0353 (4)	0.0227 (3)	0.0097 (3)	−0.0081 (3)	−0.0072 (3)
C74A	0.0177 (4)	0.0156 (3)	0.0137 (3)	0.0022 (3)	−0.0037 (3)	−0.0009 (2)
C75A	0.0201 (4)	0.0241 (4)	0.0168 (3)	0.0046 (3)	−0.0060 (3)	−0.0037 (3)
C76A	0.0223 (4)	0.0235 (4)	0.0168 (3)	0.0013 (3)	−0.0036 (3)	−0.0040 (3)
C77A	0.0225 (4)	0.0147 (3)	0.0163 (3)	0.0000 (3)	0.0009 (3)	−0.0006 (3)
C78A	0.0218 (4)	0.0165 (3)	0.0174 (3)	0.0040 (3)	−0.0007 (3)	0.0015 (3)
C79A	0.0197 (4)	0.0167 (3)	0.0161 (3)	0.0036 (3)	−0.0032 (3)	0.0008 (3)
N80A	0.0294 (4)	0.0209 (4)	0.0194 (3)	−0.0001 (3)	0.0055 (3)	−0.0028 (3)
O81A	0.0419 (6)	0.0404 (5)	0.0216 (4)	0.0039 (4)	−0.0017 (4)	−0.0120 (4)
C82A	0.0424 (5)	0.0286 (4)	0.0255 (4)	0.0128 (4)	0.0074 (4)	−0.0020 (3)
C1B	0.0230 (4)	0.0127 (3)	0.0155 (3)	0.0016 (3)	0.0038 (3)	−0.0012 (2)
C2B	0.0174 (4)	0.0161 (3)	0.0179 (3)	0.0029 (3)	0.0035 (3)	0.0013 (3)
C3B	0.0169 (4)	0.0154 (3)	0.0153 (3)	0.0020 (3)	0.0001 (3)	0.0018 (2)
C4B	0.0150 (3)	0.0178 (3)	0.0127 (3)	0.0022 (3)	0.0014 (2)	0.0008 (2)
C5B	0.0147 (3)	0.0141 (3)	0.0148 (3)	−0.0021 (2)	0.0008 (2)	0.0011 (2)
C6B	0.0112 (3)	0.0180 (3)	0.0197 (3)	−0.0028 (3)	0.0011 (3)	0.0024 (3)
C7B	0.0101 (3)	0.0169 (3)	0.0150 (3)	−0.0003 (2)	−0.0001 (2)	0.0011 (2)
C8B	0.0116 (3)	0.0138 (3)	0.0123 (3)	−0.0007 (2)	−0.0008 (2)	0.0003 (2)
C9B	0.0130 (3)	0.0109 (3)	0.0110 (3)	−0.0004 (2)	0.0000 (2)	−0.0009 (2)
C10B	0.0181 (4)	0.0115 (3)	0.0126 (3)	−0.0023 (2)	−0.0001 (2)	−0.0013 (2)
C11B	0.0174 (3)	0.0120 (3)	0.0125 (3)	−0.0007 (2)	0.0035 (2)	−0.0023 (2)
C12B	0.0142 (3)	0.0122 (3)	0.0098 (3)	−0.0007 (2)	0.0011 (2)	−0.0019 (2)
C13B	0.0140 (3)	0.0131 (3)	0.0096 (3)	−0.0006 (2)	−0.0012 (2)	−0.0004 (2)
C14B	0.0118 (3)	0.0128 (3)	0.0113 (3)	0.0006 (2)	−0.0004 (2)	0.0001 (2)
C15B	0.0134 (3)	0.0169 (3)	0.0184 (3)	0.0029 (3)	−0.0002 (3)	0.0014 (3)
C16B	0.0165 (4)	0.0156 (3)	0.0170 (3)	0.0041 (3)	−0.0016 (3)	0.0027 (3)
C17B	0.0164 (3)	0.0119 (3)	0.0106 (3)	0.0009 (2)	−0.0014 (2)	0.0005 (2)
C18B	0.0222 (4)	0.0195 (3)	0.0123 (3)	−0.0021 (3)	−0.0055 (3)	−0.0019 (3)
C19B	0.0271 (5)	0.0166 (3)	0.0173 (3)	−0.0068 (3)	−0.0042 (3)	−0.0029 (3)
C20B	0.0242 (4)	0.0148 (3)	0.0113 (3)	−0.0010 (3)	−0.0014 (3)	0.0012 (2)
C21B	0.0272 (5)	0.0188 (4)	0.0176 (4)	−0.0020 (3)	0.0060 (3)	0.0010 (3)
C22B	0.0286 (5)	0.0138 (3)	0.0151 (3)	0.0005 (3)	−0.0034 (3)	0.0033 (3)
C23B	0.0294 (5)	0.0136 (3)	0.0173 (3)	0.0007 (3)	−0.0022 (3)	0.0002 (3)
C24B	0.0330 (5)	0.0138 (3)	0.0212 (4)	−0.0027 (3)	0.0023 (4)	−0.0001 (3)
S25B	0.03778 (15)	0.01445 (9)	0.02065 (10)	0.00554 (9)	0.00050 (10)	0.00261 (7)
O31B	0.0179 (3)	0.0176 (3)	0.0178 (3)	0.0051 (2)	−0.0028 (2)	0.0015 (2)
C32B	0.0183 (4)	0.0140 (3)	0.0219 (4)	0.0026 (3)	−0.0020 (3)	0.0044 (3)
O33B	0.0303 (4)	0.0156 (3)	0.0353 (4)	0.0068 (3)	−0.0078 (3)	−0.0017 (3)
C34B	0.0159 (4)	0.0152 (3)	0.0184 (3)	0.0027 (3)	0.0006 (3)	0.0058 (3)
C35B	0.0169 (4)	0.0182 (4)	0.0261 (4)	0.0046 (3)	−0.0016 (3)	0.0014 (3)
C36B	0.0160 (4)	0.0210 (4)	0.0241 (4)	0.0019 (3)	−0.0006 (3)	0.0047 (3)
C37B	0.0190 (4)	0.0230 (4)	0.0157 (3)	0.0004 (3)	−0.0003 (3)	0.0068 (3)
C38B	0.0253 (5)	0.0307 (5)	0.0163 (4)	0.0107 (4)	−0.0020 (3)	0.0008 (3)
C39B	0.0213 (4)	0.0273 (4)	0.0165 (3)	0.0100 (3)	−0.0013 (3)	0.0021 (3)
N40B	0.0268 (5)	0.0327 (5)	0.0190 (4)	−0.0006 (4)	−0.0044 (3)	0.0044 (3)
O41B	0.0206 (4)	0.0379 (5)	0.0322 (4)	−0.0055 (3)	−0.0067 (3)	0.0055 (4)
O42B	0.0611 (9)	0.0875 (13)	0.0450 (7)	0.0320 (9)	−0.0267 (7)	−0.0368 (8)
O51B	0.0111 (2)	0.0141 (2)	0.0116 (2)	0.00058 (18)	0.00064 (18)	−0.00238 (18)
C52B	0.0127 (3)	0.0195 (3)	0.0168 (3)	0.0003 (3)	0.0017 (3)	−0.0063 (3)
O53B	0.0172 (3)	0.0474 (5)	0.0290 (4)	0.0003 (3)	0.0051 (3)	−0.0239 (4)
C54B	0.0129 (3)	0.0158 (3)	0.0156 (3)	0.0015 (2)	0.0006 (2)	−0.0037 (2)
C55B	0.0120 (4)	0.0266 (4)	0.0243 (4)	0.0017 (3)	0.0019 (3)	−0.0092 (3)
C56B	0.0125 (4)	0.0247 (4)	0.0284 (5)	0.0022 (3)	−0.0022 (3)	−0.0076 (3)
C57B	0.0160 (4)	0.0138 (3)	0.0206 (4)	0.0017 (3)	−0.0056 (3)	−0.0022 (3)
C58B	0.0185 (4)	0.0164 (3)	0.0165 (3)	0.0011 (3)	−0.0009 (3)	−0.0039 (3)
C59B	0.0144 (3)	0.0167 (3)	0.0151 (3)	0.0002 (3)	0.0011 (3)	−0.0031 (2)
N60B	0.0224 (4)	0.0190 (3)	0.0299 (4)	0.0022 (3)	−0.0114 (3)	−0.0060 (3)
O61B	0.0178 (4)	0.0519 (7)	0.0688 (9)	−0.0015 (4)	−0.0094 (5)	−0.0330 (7)
O62B	0.0420 (6)	0.0389 (5)	0.0243 (4)	−0.0104 (4)	−0.0050 (4)	−0.0119 (4)
O71B	0.0101 (2)	0.0171 (3)	0.0135 (2)	0.00167 (19)	0.00240 (19)	−0.00137 (19)
C72B	0.0133 (3)	0.0156 (3)	0.0136 (3)	0.0030 (2)	0.0039 (2)	0.0022 (2)
O73B	0.0120 (3)	0.0319 (4)	0.0219 (3)	0.0016 (3)	0.0052 (2)	−0.0005 (3)
C74B	0.0144 (3)	0.0137 (3)	0.0130 (3)	0.0029 (2)	0.0044 (2)	0.0019 (2)
C75B	0.0164 (4)	0.0222 (4)	0.0173 (3)	0.0045 (3)	0.0055 (3)	−0.0026 (3)
C76B	0.0196 (4)	0.0222 (4)	0.0158 (3)	0.0056 (3)	0.0030 (3)	−0.0026 (3)
C77B	0.0196 (4)	0.0148 (3)	0.0149 (3)	0.0028 (3)	0.0000 (3)	0.0020 (2)
C78B	0.0177 (4)	0.0171 (3)	0.0155 (3)	−0.0005 (3)	0.0027 (3)	0.0031 (3)
C79B	0.0153 (3)	0.0168 (3)	0.0135 (3)	0.0005 (3)	0.0045 (3)	0.0022 (2)
N80B	0.0276 (4)	0.0193 (3)	0.0160 (3)	0.0012 (3)	−0.0038 (3)	0.0023 (3)
O81B	0.0385 (5)	0.0314 (4)	0.0203 (3)	0.0051 (4)	−0.0048 (3)	−0.0072 (3)
O82B	0.0253 (4)	0.0365 (5)	0.0260 (4)	−0.0046 (3)	−0.0040 (3)	−0.0007 (3)
C1C	0.0187 (4)	0.0322 (5)	0.0116 (3)	0.0019 (3)	0.0010 (3)	0.0014 (3)
C2C	0.0154 (4)	0.0367 (5)	0.0150 (3)	−0.0008 (3)	0.0008 (3)	0.0023 (3)
C3C	0.0183 (4)	0.0313 (5)	0.0148 (3)	−0.0055 (3)	−0.0021 (3)	0.0057 (3)
C4C	0.0162 (4)	0.0223 (4)	0.0212 (4)	−0.0013 (3)	−0.0021 (3)	0.0034 (3)
C5C	0.0149 (4)	0.0214 (4)	0.0179 (3)	0.0009 (3)	−0.0045 (3)	0.0040 (3)
C6C	0.0125 (4)	0.0216 (4)	0.0249 (4)	0.0025 (3)	−0.0049 (3)	0.0023 (3)
C7C	0.0100 (3)	0.0189 (3)	0.0221 (4)	0.0010 (3)	−0.0009 (3)	−0.0015 (3)
C8C	0.0128 (3)	0.0163 (3)	0.0143 (3)	0.0010 (2)	0.0002 (2)	−0.0012 (2)
C9C	0.0123 (3)	0.0188 (3)	0.0105 (3)	0.0031 (2)	−0.0006 (2)	−0.0001 (2)
C10C	0.0150 (3)	0.0224 (4)	0.0113 (3)	0.0026 (3)	−0.0020 (2)	0.0018 (3)
C11C	0.0188 (4)	0.0211 (4)	0.0107 (3)	0.0068 (3)	−0.0004 (3)	−0.0020 (3)
C12C	0.0210 (4)	0.0152 (3)	0.0109 (3)	0.0041 (3)	−0.0020 (3)	−0.0035 (2)
C13C	0.0226 (4)	0.0140 (3)	0.0125 (3)	−0.0017 (3)	−0.0004 (3)	−0.0028 (2)
C14C	0.0166 (4)	0.0149 (3)	0.0146 (3)	−0.0007 (3)	0.0030 (3)	−0.0019 (2)
C15C	0.0190 (4)	0.0228 (4)	0.0224 (4)	−0.0030 (3)	0.0072 (3)	−0.0021 (3)
C16C	0.0278 (5)	0.0241 (4)	0.0196 (4)	−0.0086 (4)	0.0072 (3)	−0.0011 (3)
C17C	0.0293 (5)	0.0149 (3)	0.0138 (3)	−0.0056 (3)	0.0014 (3)	−0.0017 (3)
C18C	0.0283 (5)	0.0197 (4)	0.0185 (4)	−0.0037 (3)	−0.0037 (3)	−0.0051 (3)
C19C	0.0243 (5)	0.0270 (4)	0.0140 (3)	0.0016 (3)	−0.0065 (3)	−0.0011 (3)
C20C	0.0469 (7)	0.0158 (4)	0.0176 (4)	−0.0081 (4)	−0.0027 (4)	−0.0004 (3)
C21C	0.0614 (9)	0.0182 (4)	0.0225 (5)	0.0067 (5)	0.0017 (5)	0.0010 (3)
C22C	0.0434 (7)	0.0231 (4)	0.0199 (4)	−0.0150 (4)	−0.0019 (4)	0.0042 (3)
C23C	0.0348 (6)	0.0263 (5)	0.0150 (4)	−0.0085 (4)	0.0018 (4)	0.0020 (3)
C24C	0.0406 (6)	0.0279 (5)	0.0166 (4)	−0.0113 (4)	−0.0010 (4)	0.0065 (3)
S25C	0.03528 (14)	0.02368 (11)	0.01677 (9)	−0.00023 (10)	−0.00252 (9)	0.00354 (8)
O31C	0.0214 (3)	0.0360 (4)	0.0167 (3)	−0.0112 (3)	−0.0009 (3)	0.0062 (3)
C32C	0.0170 (4)	0.0224 (4)	0.0177 (4)	−0.0001 (3)	0.0009 (3)	0.0066 (3)
O33C	0.0347 (5)	0.0430 (5)	0.0172 (3)	−0.0128 (4)	0.0008 (3)	0.0079 (3)
C34C	0.0144 (3)	0.0172 (3)	0.0168 (3)	0.0018 (3)	0.0029 (3)	0.0055 (3)
C35C	0.0201 (4)	0.0214 (4)	0.0178 (4)	−0.0024 (3)	0.0056 (3)	0.0051 (3)
C36C	0.0196 (4)	0.0197 (4)	0.0199 (4)	−0.0032 (3)	0.0063 (3)	0.0031 (3)
C37C	0.0145 (3)	0.0150 (3)	0.0188 (3)	0.0006 (3)	0.0040 (3)	0.0021 (3)
C38C	0.0146 (3)	0.0187 (3)	0.0175 (3)	0.0008 (3)	0.0036 (3)	0.0050 (3)
C39C	0.0131 (3)	0.0194 (3)	0.0175 (3)	0.0004 (3)	0.0023 (3)	0.0064 (3)
N40C	0.0184 (4)	0.0197 (3)	0.0214 (3)	−0.0019 (3)	0.0054 (3)	−0.0014 (3)
O41C	0.0345 (5)	0.0363 (5)	0.0301 (4)	−0.0198 (4)	0.0059 (4)	−0.0002 (4)
O42C	0.0227 (4)	0.0285 (4)	0.0187 (3)	−0.0015 (3)	0.0031 (3)	−0.0006 (3)
O51C	0.0190 (3)	0.0152 (2)	0.0112 (2)	0.0045 (2)	−0.0023 (2)	−0.00312 (19)
C52C	0.0218 (4)	0.0221 (4)	0.0147 (3)	0.0087 (3)	−0.0034 (3)	−0.0066 (3)
O53C	0.0292 (5)	0.0513 (6)	0.0316 (4)	0.0202 (4)	−0.0094 (4)	−0.0296 (5)
C54C	0.0188 (4)	0.0159 (3)	0.0117 (3)	0.0066 (3)	−0.0013 (3)	−0.0029 (2)
C55C	0.0197 (4)	0.0213 (4)	0.0134 (3)	0.0073 (3)	0.0017 (3)	−0.0026 (3)
C56C	0.0168 (4)	0.0185 (3)	0.0141 (3)	0.0037 (3)	0.0009 (3)	0.0001 (3)
C57C	0.0183 (4)	0.0130 (3)	0.0116 (3)	0.0022 (2)	−0.0012 (2)	0.0006 (2)
C58C	0.0191 (4)	0.0171 (3)	0.0107 (3)	0.0032 (3)	0.0006 (2)	−0.0018 (2)
C59C	0.0174 (4)	0.0194 (3)	0.0125 (3)	0.0042 (3)	0.0000 (3)	−0.0029 (3)
N60C	0.0230 (4)	0.0147 (3)	0.0145 (3)	−0.0018 (3)	−0.0021 (3)	0.0010 (2)
O61C	0.0225 (4)	0.0342 (4)	0.0293 (4)	−0.0073 (3)	−0.0018 (3)	−0.0044 (3)
O62C	0.0311 (4)	0.0214 (3)	0.0159 (3)	0.0020 (3)	−0.0021 (3)	−0.0050 (2)
O71C	0.0110 (3)	0.0192 (3)	0.0213 (3)	0.0019 (2)	0.0017 (2)	−0.0041 (2)
C72C	0.0131 (3)	0.0123 (3)	0.0232 (4)	0.0025 (2)	0.0028 (3)	0.0001 (3)
O73C	0.0117 (3)	0.0251 (4)	0.0407 (5)	0.0019 (2)	0.0042 (3)	−0.0093 (3)
C74C	0.0142 (3)	0.0122 (3)	0.0205 (4)	0.0020 (2)	0.0038 (3)	0.0004 (3)
C75C	0.0171 (4)	0.0206 (4)	0.0278 (5)	0.0023 (3)	0.0061 (3)	−0.0061 (3)
C76C	0.0222 (4)	0.0208 (4)	0.0275 (5)	0.0013 (3)	0.0058 (4)	−0.0067 (3)
C77C	0.0215 (4)	0.0144 (3)	0.0230 (4)	−0.0005 (3)	0.0007 (3)	−0.0011 (3)
C78C	0.0165 (4)	0.0244 (4)	0.0257 (4)	−0.0008 (3)	0.0021 (3)	−0.0030 (3)
C79C	0.0141 (4)	0.0251 (4)	0.0242 (4)	−0.0001 (3)	0.0036 (3)	−0.0050 (3)
N80C	0.0298 (5)	0.0226 (4)	0.0292 (5)	−0.0031 (3)	−0.0021 (4)	−0.0051 (3)
O81C	0.0400 (6)	0.0638 (9)	0.0531 (8)	−0.0040 (6)	0.0018 (6)	−0.0390 (7)
O82C	0.0275 (4)	0.0251 (4)	0.0420 (5)	−0.0027 (3)	−0.0079 (4)	−0.0048 (4)
C1D	0.0210 (4)	0.0235 (4)	0.0132 (3)	0.0039 (3)	0.0004 (3)	−0.0006 (3)
C2D	0.0177 (4)	0.0238 (4)	0.0166 (3)	0.0048 (3)	0.0006 (3)	0.0006 (3)
C3D	0.0209 (4)	0.0214 (4)	0.0165 (3)	0.0078 (3)	0.0045 (3)	0.0027 (3)
C4D	0.0185 (4)	0.0161 (3)	0.0222 (4)	0.0044 (3)	0.0045 (3)	0.0014 (3)
C5D	0.0187 (4)	0.0160 (3)	0.0178 (3)	0.0036 (3)	0.0071 (3)	0.0032 (3)
C6D	0.0170 (4)	0.0150 (3)	0.0247 (4)	0.0005 (3)	0.0090 (3)	0.0045 (3)
C7D	0.0125 (3)	0.0123 (3)	0.0224 (4)	−0.0006 (2)	0.0059 (3)	0.0002 (3)
C8D	0.0105 (3)	0.0118 (3)	0.0172 (3)	0.0002 (2)	0.0037 (2)	0.0003 (2)
C9D	0.0112 (3)	0.0136 (3)	0.0129 (3)	0.0003 (2)	0.0037 (2)	−0.0005 (2)
C10D	0.0178 (4)	0.0170 (3)	0.0127 (3)	0.0030 (3)	0.0052 (3)	0.0006 (2)
C11D	0.0135 (3)	0.0159 (3)	0.0116 (3)	−0.0025 (2)	0.0011 (2)	−0.0014 (2)
C12D	0.0108 (3)	0.0129 (3)	0.0117 (3)	−0.0021 (2)	0.0017 (2)	−0.0025 (2)
C13D	0.0103 (3)	0.0108 (3)	0.0139 (3)	−0.0006 (2)	0.0005 (2)	−0.0017 (2)
C14D	0.0098 (3)	0.0110 (3)	0.0178 (3)	−0.0011 (2)	0.0005 (2)	−0.0014 (2)
C15D	0.0115 (3)	0.0161 (3)	0.0276 (4)	−0.0028 (3)	−0.0035 (3)	0.0006 (3)
C16D	0.0139 (4)	0.0175 (3)	0.0226 (4)	0.0002 (3)	−0.0056 (3)	−0.0009 (3)
C17D	0.0132 (3)	0.0127 (3)	0.0150 (3)	0.0002 (2)	−0.0011 (2)	−0.0016 (2)
C18D	0.0159 (4)	0.0134 (3)	0.0189 (3)	0.0013 (2)	0.0043 (3)	−0.0029 (3)
C19D	0.0265 (5)	0.0210 (4)	0.0163 (3)	0.0039 (3)	0.0105 (3)	−0.0010 (3)
C20D	0.0223 (4)	0.0128 (3)	0.0176 (3)	0.0012 (3)	−0.0004 (3)	0.0006 (3)
C21D	0.0285 (5)	0.0172 (4)	0.0227 (4)	−0.0060 (3)	−0.0002 (4)	0.0019 (3)
C22D	0.0288 (5)	0.0194 (4)	0.0196 (4)	0.0050 (3)	−0.0032 (3)	0.0025 (3)
C23D	0.0353 (6)	0.0250 (4)	0.0159 (4)	0.0053 (4)	−0.0018 (4)	0.0029 (3)
C24D	0.0527 (8)	0.0323 (6)	0.0194 (4)	0.0131 (6)	−0.0067 (5)	0.0045 (4)
S25D	0.0607 (2)	0.02159 (11)	0.01541 (10)	−0.00607 (12)	−0.00009 (11)	0.00603 (8)
O31D	0.0233 (3)	0.0235 (3)	0.0170 (3)	0.0117 (3)	0.0024 (2)	0.0027 (2)
C32D	0.0177 (4)	0.0210 (4)	0.0197 (4)	0.0055 (3)	−0.0020 (3)	0.0004 (3)
O33D	0.0382 (5)	0.0464 (6)	0.0217 (4)	0.0217 (5)	−0.0115 (4)	−0.0095 (4)
C34D	0.0130 (3)	0.0152 (3)	0.0181 (3)	0.0022 (2)	−0.0026 (3)	0.0013 (3)
C35D	0.0184 (4)	0.0207 (4)	0.0218 (4)	0.0057 (3)	−0.0091 (3)	−0.0038 (3)
C36D	0.0174 (4)	0.0198 (4)	0.0219 (4)	0.0057 (3)	−0.0087 (3)	−0.0030 (3)
C37D	0.0142 (3)	0.0133 (3)	0.0186 (3)	0.0014 (2)	−0.0022 (3)	0.0007 (2)
C38D	0.0179 (4)	0.0181 (3)	0.0164 (3)	0.0039 (3)	−0.0035 (3)	0.0029 (3)
C39D	0.0155 (4)	0.0183 (3)	0.0173 (3)	0.0040 (3)	−0.0025 (3)	0.0037 (3)
N40D	0.0229 (4)	0.0194 (3)	0.0224 (4)	0.0065 (3)	−0.0044 (3)	−0.0017 (3)
O41D	0.0518 (7)	0.0401 (5)	0.0292 (4)	0.0324 (5)	−0.0152 (4)	−0.0074 (4)
O42D	0.0470 (6)	0.0304 (4)	0.0207 (4)	0.0169 (4)	0.0009 (4)	−0.0002 (3)
O51D	0.0096 (2)	0.0153 (2)	0.0125 (2)	−0.00187 (18)	0.00210 (18)	−0.00295 (18)
C52D	0.0112 (3)	0.0210 (4)	0.0184 (3)	−0.0040 (3)	0.0030 (3)	−0.0075 (3)
O53D	0.0169 (4)	0.0467 (6)	0.0480 (6)	−0.0150 (4)	0.0119 (4)	−0.0352 (5)
C54D	0.0101 (3)	0.0158 (3)	0.0138 (3)	−0.0016 (2)	0.0005 (2)	−0.0028 (2)
C55D	0.0113 (3)	0.0228 (4)	0.0181 (3)	−0.0036 (3)	0.0022 (3)	−0.0079 (3)
C56D	0.0110 (3)	0.0223 (4)	0.0171 (3)	−0.0006 (3)	0.0013 (3)	−0.0056 (3)
C57D	0.0134 (3)	0.0144 (3)	0.0134 (3)	0.0028 (2)	−0.0004 (2)	−0.0015 (2)
C58D	0.0140 (3)	0.0131 (3)	0.0153 (3)	0.0002 (2)	−0.0014 (2)	−0.0017 (2)
C59D	0.0114 (3)	0.0146 (3)	0.0154 (3)	−0.0015 (2)	−0.0003 (2)	−0.0019 (2)
N60D	0.0175 (3)	0.0176 (3)	0.0154 (3)	0.0050 (2)	−0.0001 (2)	−0.0022 (2)
O61D	0.0170 (3)	0.0364 (4)	0.0266 (4)	0.0060 (3)	0.0027 (3)	−0.0103 (3)
O62D	0.0260 (4)	0.0230 (3)	0.0241 (3)	0.0019 (3)	−0.0011 (3)	−0.0109 (3)
O71D	0.0123 (3)	0.0130 (2)	0.0226 (3)	−0.00135 (19)	0.0031 (2)	−0.0028 (2)
C72D	0.0129 (3)	0.0129 (3)	0.0186 (3)	−0.0002 (2)	−0.0006 (3)	0.0029 (2)
O73D	0.0132 (3)	0.0253 (3)	0.0275 (4)	−0.0038 (2)	0.0000 (3)	−0.0016 (3)
C74D	0.0146 (3)	0.0138 (3)	0.0168 (3)	0.0002 (2)	−0.0013 (3)	0.0026 (2)
C75D	0.0157 (4)	0.0232 (4)	0.0224 (4)	0.0017 (3)	−0.0051 (3)	−0.0038 (3)
C76D	0.0220 (4)	0.0248 (4)	0.0211 (4)	0.0041 (3)	−0.0064 (3)	−0.0044 (3)
C77D	0.0241 (4)	0.0162 (3)	0.0160 (3)	0.0047 (3)	0.0000 (3)	0.0023 (3)
C78D	0.0198 (4)	0.0176 (3)	0.0233 (4)	−0.0030 (3)	0.0052 (3)	−0.0006 (3)
C79D	0.0174 (4)	0.0168 (3)	0.0213 (4)	−0.0043 (3)	0.0031 (3)	−0.0016 (3)
N80D	0.0323 (5)	0.0191 (3)	0.0184 (3)	0.0078 (3)	0.0018 (3)	0.0019 (3)
O81D	0.0440 (6)	0.0321 (4)	0.0234 (4)	0.0133 (4)	−0.0041 (4)	−0.0079 (3)
O82D	0.0319 (5)	0.0317 (4)	0.0303 (4)	0.0051 (4)	0.0105 (4)	−0.0007 (3)
C101	0.0252 (5)	0.0211 (4)	0.0339 (5)	−0.0008 (4)	0.0011 (4)	−0.0011 (4)
C102	0.0172 (4)	0.0201 (4)	0.0251 (4)	−0.0012 (3)	−0.0022 (3)	0.0043 (3)
O103	0.0228 (4)	0.0334 (5)	0.0741 (9)	0.0032 (4)	−0.0197 (5)	−0.0050 (5)
O104	0.0145 (3)	0.0159 (3)	0.0418 (5)	0.0009 (2)	−0.0022 (3)	−0.0001 (3)
C105	0.0178 (4)	0.0153 (4)	0.0538 (8)	−0.0008 (3)	0.0093 (5)	0.0026 (4)
C106	0.0172 (4)	0.0218 (4)	0.0537 (8)	−0.0041 (3)	0.0052 (5)	−0.0001 (5)

### Geometric parameters (Å, °)

**Table:**

C1A—C2A	1.5262 (18)	C11C—C12C	1.5360 (18)
C1A—C10A	1.5439 (19)	C11C—H11C1	0.9900
C1A—H1A1	0.9900	C11C—H11C2	0.9900
C1A—H1A2	0.9900	C12C—O51C	1.4652 (14)
C2A—C3A	1.5179 (18)	C12C—C13C	1.5307 (19)
C2A—H2A1	0.9900	C12C—H12C1	1.0000
C2A—H2A2	0.9900	C13C—C18C	1.5427 (16)
C3A—O31A	1.4694 (16)	C13C—C14C	1.5460 (19)
C3A—C4A	1.514 (2)	C13C—C17C	1.5575 (19)
C3A—H3A1	1.0000	C14C—C15C	1.5329 (18)
C4A—C5A	1.5435 (17)	C14C—H14C1	1.0000
C4A—H4A1	0.9900	C15C—C16C	1.543 (2)
C4A—H4A2	0.9900	C15C—H15C1	0.9900
C5A—C6A	1.531 (2)	C15C—H15C2	0.9900
C5A—C10A	1.5499 (18)	C16C—C17C	1.554 (2)
C5A—H5A1	1.0000	C16C—H16C1	0.9900
C6A—C7A	1.5262 (19)	C16C—H16C2	0.9900
C6A—H6A1	0.9900	C17C—C20C	1.539 (2)
C6A—H6A2	0.9900	C17C—H17C1	1.0000
C7A—O71A	1.4676 (15)	C18C—H18C1	0.9800
C7A—C8A	1.5273 (16)	C18C—H18C2	0.9800
C7A—H7A1	1.0000	C18C—H18C3	0.9800
C8A—C14A	1.5317 (19)	C19C—H19C1	0.9800
C8A—C9A	1.5487 (18)	C19C—H19C2	0.9800
C8A—H8A1	1.0000	C19C—H19C3	0.9800
C9A—C11A	1.5340 (17)	C20C—C21C	1.523 (3)
C9A—C10A	1.5594 (19)	C20C—C22C	1.543 (2)
C9A—H9A1	1.0000	C20C—H20C1	1.0000
C10A—C19A	1.5434 (16)	C21C—H21C1	0.9800
C11A—C12A	1.5274 (19)	C21C—H21C2	0.9800
C11A—H11A1	0.9900	C21C—H21C3	0.9800
C11A—H11A2	0.9900	C22C—C23C	1.5272 (19)
C12A—O51A	1.4665 (14)	C22C—H22C1	0.9900
C12A—C13A	1.5305 (18)	C22C—H22C2	0.9900
C12A—H12A1	1.0000	C23C—C24C	1.517 (2)
C13A—C18A	1.5448 (16)	C23C—H23C1	0.9900
C13A—C14A	1.5492 (17)	C23C—H23C2	0.9900
C13A—C17A	1.5497 (19)	C24C—S25C	1.8140 (17)
C14A—C15A	1.5361 (17)	C24C—H24C1	0.9900
C14A—H14A1	1.0000	C24C—H24C2	0.9900
C15A—C16A	1.5515 (18)	S25C—S25D	2.031 (2)
C15A—H15A1	0.9900	O31C—C32C	1.3308 (15)
C15A—H15A2	0.9900	C32C—O33C	1.2053 (17)
C16A—C17A	1.5545 (18)	C32C—C34C	1.4971 (17)
C16A—H16A1	0.9900	C34C—C35C	1.3958 (16)
C16A—H16A2	0.9900	C34C—C39C	1.3967 (19)
C17A—C20A	1.5399 (17)	C35C—C36C	1.3914 (18)
C17A—H17A1	1.0000	C35C—H35C1	0.9500
C18A—H18A1	0.9800	C36C—C37C	1.3863 (19)
C18A—H18A2	0.9800	C36C—H36C1	0.9500
C18A—H18A3	0.9800	C37C—C38C	1.3896 (16)
C19A—H19A1	0.9800	C37C—N40C	1.4675 (17)
C19A—H19A2	0.9800	C38C—C39C	1.3886 (17)
C19A—H19A3	0.9800	C38C—H38C1	0.9500
C20A—C21A	1.531 (2)	C39C—H39C1	0.9500
C20A—C22A	1.544 (2)	N40C—O41C	1.2226 (15)
C20A—H20A1	1.0000	N40C—O42C	1.2271 (17)
C21A—H21A1	0.9800	O51C—C52C	1.3317 (17)
C21A—H21A2	0.9800	C52C—O53C	1.2074 (14)
C21A—H21A3	0.9800	C52C—C54C	1.4975 (17)
C22A—C23A	1.5304 (18)	C54C—C59C	1.3962 (16)
C22A—H22A1	0.9900	C54C—C55C	1.399 (2)
C22A—H22A2	0.9900	C55C—C56C	1.3904 (16)
C23A—C24A	1.520 (2)	C55C—H55C1	0.9500
C23A—H23A1	0.9900	C56C—C57C	1.3870 (16)
C23A—H23A2	0.9900	C56C—H56C1	0.9500
C24A—S25A	1.8187 (17)	C57C—C58C	1.3864 (19)
C24A—H24A1	0.9900	C57C—N60C	1.4680 (15)
C24A—H24A2	0.9900	C58C—C59C	1.3939 (16)
S25A—S25B	2.0388 (18)	C58C—H58C1	0.9500
O31A—C32A	1.3377 (17)	C59C—H59C1	0.9500
C32A—O33A	1.2121 (16)	N60C—O61C	1.2193 (18)
C32A—C34A	1.4972 (18)	N60C—O62C	1.2305 (14)
C34A—C35A	1.3933 (18)	O71C—C72C	1.3331 (14)
C34A—C39A	1.3996 (18)	C72C—O73C	1.2099 (17)
C35A—C36A	1.3864 (18)	C72C—C74C	1.4978 (16)
C35A—H35A1	0.9500	C74C—C75C	1.3925 (16)
C36A—C37A	1.3837 (18)	C74C—C79C	1.3942 (19)
C36A—H36A1	0.9500	C75C—C76C	1.3885 (18)
C37A—C38A	1.3897 (19)	C75C—H75C1	0.9500
C37A—N40A	1.4722 (17)	C76C—C77C	1.384 (2)
C38A—C39A	1.3900 (18)	C76C—H76C1	0.9500
C38A—H38A1	0.9500	C77C—C78C	1.3814 (17)
C39A—H39A1	0.9500	C77C—N80C	1.4788 (12)
N40A—O41A	1.2211 (17)	C78C—C79C	1.3886 (18)
N40A—O42A	1.2268 (19)	C78C—H78C1	0.9500
O51A—C52A	1.3348 (17)	C79C—H79C1	0.9500
C52A—O53A	1.2096 (15)	N80C—O81C	1.2204 (11)
C52A—C54A	1.5000 (17)	N80C—O82C	1.2215 (14)
C54A—C55A	1.396 (2)	C1D—C2D	1.5273 (17)
C54A—C59A	1.3981 (16)	C1D—C10D	1.549 (2)
C55A—C56A	1.385 (2)	C1D—H1D1	0.9900
C55A—H55A1	0.9500	C1D—H1D2	0.9900
C56A—C57A	1.386 (2)	C2D—C3D	1.512 (2)
C56A—H56A1	0.9500	C2D—H2D1	0.9900
C57A—C58A	1.388 (2)	C2D—H2D2	0.9900
C57A—N60A	1.4729 (19)	C3D—O31D	1.4636 (15)
C58A—C59A	1.3890 (17)	C3D—C4D	1.515 (2)
C58A—H58A1	0.9500	C3D—H3D1	1.0000
C59A—H59A1	0.9500	C4D—C5D	1.5377 (16)
N60A—C62A	1.222 (2)	C4D—H4D1	0.9900
N60A—O61A	1.2283 (19)	C4D—H4D2	0.9900
O71A—C72A	1.3376 (15)	C5D—C6D	1.537 (2)
C72A—O73A	1.2135 (16)	C5D—C10D	1.5540 (19)
C72A—C74A	1.4921 (16)	C5D—H5D1	1.0000
C74A—C79A	1.3929 (17)	C6D—C7D	1.5225 (19)
C74A—C75A	1.3979 (17)	C6D—H6D1	0.9900
C75A—C76A	1.3853 (17)	C6D—H6D2	0.9900
C75A—H75A1	0.9500	C7D—O71D	1.4641 (14)
C76A—C77A	1.3857 (18)	C7D—C8D	1.5264 (18)
C76A—H76A1	0.9500	C7D—H7D1	1.0000
C77A—C78A	1.3848 (18)	C8D—C14D	1.5224 (18)
C77A—N80A	1.4686 (17)	C8D—C9D	1.5431 (18)
C78A—C79A	1.3935 (17)	C8D—H8D1	1.0000
C78A—H78A1	0.9500	C9D—C11D	1.5397 (18)
C79A—H79A1	0.9500	C9D—C10D	1.5595 (18)
N80A—O81A	1.2239 (18)	C9D—H9D1	1.0000
N80A—C82A	1.2242 (17)	C10D—C19D	1.5407 (16)
C1B—C2B	1.5268 (18)	C11D—C12D	1.5363 (17)
C1B—C10B	1.5392 (18)	C11D—H11D1	0.9900
C1B—H1B1	0.9900	C11D—H11D2	0.9900
C1B—H1B2	0.9900	C12D—O51D	1.4652 (13)
C2B—C3B	1.5141 (17)	C12D—C13D	1.5347 (18)
C2B—H2B1	0.9900	C12D—H12D1	1.0000
C2B—H2B2	0.9900	C13D—C18D	1.5397 (15)
C3B—O31B	1.4608 (16)	C13D—C14D	1.5427 (18)
C3B—C4B	1.5171 (17)	C13D—C17D	1.5605 (18)
C3B—H3B1	1.0000	C14D—C15D	1.5266 (18)
C4B—C5B	1.5402 (17)	C14D—H14D1	1.0000
C4B—H4B1	0.9900	C15D—C16D	1.5444 (18)
C4B—H4B2	0.9900	C15D—H15D1	0.9900
C5B—C6B	1.5362 (17)	C15D—H15D2	0.9900
C5B—C10B	1.5512 (17)	C16D—C17D	1.5561 (18)
C5B—H5B1	1.0000	C16D—H16D1	0.9900
C6B—C7B	1.5244 (19)	C16D—H16D2	0.9900
C6B—H6B1	0.9900	C17D—C20D	1.5404 (19)
C6B—H6B2	0.9900	C17D—H17D1	1.0000
C7B—O71B	1.4582 (14)	C18D—H18D1	0.9800
C7B—C8B	1.5243 (17)	C18D—H18D2	0.9800
C7B—H7B1	1.0000	C18D—H18D3	0.9800
C8B—C14B	1.5226 (18)	C19D—H19D1	0.9800
C8B—C9B	1.5452 (17)	C19D—H19D2	0.9800
C8B—H8B1	1.0000	C19D—H19D3	0.9800
C9B—C11B	1.5381 (16)	C20D—C21D	1.5291 (19)
C9B—C10B	1.5584 (19)	C20D—C22D	1.542 (2)
C9B—H9B1	1.0000	C20D—H20D1	1.0000
C10B—C19B	1.5449 (16)	C21D—H21D1	0.9800
C11B—C12B	1.5316 (19)	C21D—H21D2	0.9800
C11B—H11B1	0.9900	C21D—H21D3	0.9800
C11B—H11B2	0.9900	C22D—C23D	1.534 (2)
C12B—O51B	1.4617 (14)	C22D—H22D1	0.9900
C12B—C13B	1.5339 (17)	C22D—H22D2	0.9900
C12B—H12B1	1.0000	C23D—C24D	1.520 (2)
C13B—C18B	1.5368 (16)	C23D—H23D1	0.9900
C13B—C14B	1.5453 (16)	C23D—H23D2	0.9900
C13B—C17B	1.5590 (19)	C24D—S25D	1.830 (2)
C14B—C15B	1.5329 (16)	C24D—H24D1	0.9900
C14B—H14B1	1.0000	C24D—H24D2	0.9900
C15B—C16B	1.5489 (17)	O31D—C32D	1.3338 (15)
C15B—H15B1	0.9900	C32D—O33D	1.2062 (18)
C15B—H15B2	0.9900	C32D—C34D	1.4962 (17)
C16B—C17B	1.5522 (18)	C34D—C35D	1.3920 (16)
C16B—H16B1	0.9900	C34D—C39D	1.3967 (18)
C16B—H16B2	0.9900	C35D—C36D	1.3887 (17)
C17B—C20B	1.5349 (16)	C35D—H35D1	0.9500
C17B—H17B1	1.0000	C36D—C37D	1.3833 (18)
C18B—H18B1	0.9800	C36D—H36D1	0.9500
C18B—H18B2	0.9800	C37D—C38D	1.3886 (16)
C18B—H18B3	0.9800	C37D—N40D	1.4663 (16)
C19B—H19B1	0.9800	C38D—C39D	1.3847 (16)
C19B—H19B2	0.9800	C38D—H38D1	0.9500
C19B—H19B3	0.9800	C39D—H39D1	0.9500
C20B—C21B	1.531 (2)	N40D—O41D	1.2229 (15)
C20B—C22B	1.546 (2)	N40D—O42D	1.2264 (18)
C20B—H20B1	1.0000	O51D—C52D	1.3366 (16)
C21B—H21B1	0.9800	C52D—O53D	1.2085 (14)
C21B—H21B2	0.9800	C52D—C54D	1.4923 (15)
C21B—H21B3	0.9800	C54D—C55D	1.3960 (18)
C22B—C23B	1.5284 (18)	C54D—C59D	1.3967 (15)
C22B—H22B1	0.9900	C55D—C56D	1.3912 (15)
C22B—H22B2	0.9900	C55D—H55D1	0.9500
C23B—C24B	1.521 (2)	C56D—C57D	1.3825 (15)
C23B—H23B1	0.9900	C56D—H56D1	0.9500
C23B—H23B2	0.9900	C57D—C58D	1.3891 (18)
C24B—S25B	1.8234 (18)	C57D—N60D	1.4731 (15)
C24B—H24B1	0.9900	C58D—C59D	1.3888 (15)
C24B—H24B2	0.9900	C58D—H58D1	0.9500
O31B—C32B	1.3354 (15)	C59D—H59D1	0.9500
C32B—O33B	1.2106 (16)	N60D—O61D	1.2158 (17)
C32B—C34B	1.4948 (18)	N60D—O62D	1.2298 (14)
C34B—C35B	1.3947 (18)	O71D—C72D	1.3326 (14)
C34B—C39B	1.3947 (17)	C72D—O73D	1.2132 (16)
C35B—C36B	1.3872 (18)	C72D—C74D	1.4946 (16)
C35B—H35B1	0.9500	C74D—C75D	1.3923 (16)
C36B—C37B	1.3794 (18)	C74D—C79D	1.3980 (19)
C36B—H36B1	0.9500	C75D—C76D	1.3971 (18)
C37B—C38B	1.3880 (19)	C75D—H75D1	0.9500
C37B—N40B	1.4723 (18)	C76D—C77D	1.382 (2)
C38B—C39B	1.3901 (19)	C76D—H76D1	0.9500
C38B—H38B1	0.9500	C77D—C78D	1.3860 (17)
C39B—H39B1	0.9500	C77D—N80D	1.4671 (17)
N40B—O41B	1.2175 (18)	C78D—C79D	1.3888 (17)
N40B—O42B	1.219 (2)	C78D—H78D1	0.9500
O51B—C52B	1.3364 (17)	C79D—H79D1	0.9500
C52B—O53B	1.2081 (14)	N80D—O81D	1.2232 (17)
C52B—C54B	1.4984 (16)	N80D—O82D	1.225 (2)
C54B—C59B	1.3920 (15)	C101—C102	1.504 (2)
C54B—C55B	1.4009 (19)	C101—H101A	0.9800
C55B—C56B	1.3938 (17)	C101—H101B	0.9800
C55B—H55B1	0.9500	C101—H101C	0.9800
C56B—C57B	1.3803 (17)	C102—O103	1.2032 (18)
C56B—H56B1	0.9500	C102—O104	1.3221 (16)
C57B—C58B	1.3853 (19)	O104—C105	1.4445 (19)
C57B—N60B	1.4755 (16)	C105—C106	1.496 (2)
C58B—C59B	1.3933 (15)	C105—H105A	0.9900
C58B—H58B1	0.9500	C105—H105B	0.9900
C59B—H59B1	0.9500	C106—H106A	0.9800
N60B—O61B	1.2199 (19)	C106—H106B	0.9800
N60B—O62B	1.2256 (18)	C106—H106C	0.9800
O71B—C72B	1.3332 (14)	C201—C202	1.5326 (13)
C72B—O73B	1.2114 (16)	C201—H201A	0.9800
C72B—C74B	1.4936 (16)	C201—H201B	0.9800
C74B—C75B	1.3909 (16)	C201—H201C	0.9800
C74B—C79B	1.3967 (18)	C202—O203	1.2284 (14)
C75B—C76B	1.3862 (17)	C202—O204	1.3572 (13)
C75B—H75B1	0.9500	O204—C205	1.4324 (13)
C76B—C77B	1.3851 (19)	C205—C206	1.5329 (16)
C76B—H76B1	0.9500	C205—H205A	0.9900
C77B—C78B	1.3872 (17)	C205—H205B	0.9900
C77B—N80B	1.4715 (16)	C206—H206A	0.9800
C78B—C79B	1.3924 (16)	C206—H206B	0.9800
C78B—H78B1	0.9500	C206—H206C	0.9800
C79B—H79B1	0.9500	C211—C212	1.5301 (13)
N80B—O81B	1.2271 (16)	C211—H211A	0.9800
N80B—O82B	1.2273 (19)	C211—H211B	0.9800
C1C—C2C	1.5273 (19)	C211—H211C	0.9800
C1C—C10C	1.5433 (19)	C212—O213	1.2275 (12)
C1C—H1C1	0.9900	C212—O214	1.3620 (16)
C1C—H1C2	0.9900	O214—C215	1.4349 (14)
C2C—C3C	1.518 (2)	C215—C216	1.5298 (14)
C2C—H2C1	0.9900	C215—H215A	0.9900
C2C—H2C2	0.9900	C215—H215B	0.9900
C3C—O31C	1.4721 (16)	C216—H216A	0.9800
C3C—C4C	1.5112 (19)	C216—H216B	0.9800
C3C—H3C1	1.0000	C216—H216C	0.9800
C4C—C5C	1.5379 (17)	C301—C302	1.5294 (15)
C4C—H4C1	0.9900	C301—H301A	0.9800
C4C—H4C2	0.9900	C301—H301B	0.9800
C5C—C6C	1.5331 (19)	C301—H301C	0.9800
C5C—C10C	1.5540 (19)	C302—C303	1.5307 (16)
C5C—H5C1	1.0000	C302—H302A	0.9900
C6C—C7C	1.5270 (19)	C302—H302B	0.9900
C6C—H6C1	0.9900	C303—C304	1.5287 (14)
C6C—H6C2	0.9900	C303—H303A	0.9900
C7C—O71C	1.4660 (15)	C303—H303B	0.9900
C7C—C8C	1.5282 (18)	C304—C305	1.5276 (16)
C7C—H7C1	1.0000	C304—H304A	0.9900
C8C—C14C	1.5182 (18)	C304—H304B	0.9900
C8C—C9C	1.5424 (18)	C305—C306	1.5299 (14)
C8C—H8C1	1.0000	C305—H305A	0.9900
C9C—C11C	1.5405 (18)	C305—H305B	0.9900
C9C—C10C	1.5620 (18)	C306—H306A	0.9800
C9C—H9C1	1.0000	C306—H306B	0.9800
C10C—C19C	1.5403 (17)	C306—H306C	0.9800
			
C2A—C1A—C10A	114.14 (9)	C11C—C9C—H9C1	107.3
C2A—C1A—H1A1	108.7	C8C—C9C—H9C1	107.3
C10A—C1A—H1A1	108.7	C10C—C9C—H9C1	107.3
C2A—C1A—H1A2	108.7	C19C—C10C—C1C	105.83 (10)
C10A—C1A—H1A2	108.7	C19C—C10C—C5C	109.85 (9)
H1A1—C1A—H1A2	107.6	C1C—C10C—C5C	107.57 (9)
C3A—C2A—C1A	109.45 (11)	C19C—C10C—C9C	111.14 (10)
C3A—C2A—H2A1	109.8	C1C—C10C—C9C	113.43 (8)
C1A—C2A—H2A1	109.8	C5C—C10C—C9C	108.91 (8)
C3A—C2A—H2A2	109.8	C12C—C11C—C9C	115.86 (8)
C1A—C2A—H2A2	109.8	C12C—C11C—H11C1	108.3
H2A1—C2A—H2A2	108.2	C9C—C11C—H11C1	108.3
O31A—C3A—C4A	105.55 (9)	C12C—C11C—H11C2	108.3
O31A—C3A—C2A	110.72 (10)	C9C—C11C—H11C2	108.3
C4A—C3A—C2A	111.70 (8)	H11C1—C11C—H11C2	107.4
O31A—C3A—H3A1	109.6	O51C—C12C—C13C	107.54 (9)
C4A—C3A—H3A1	109.6	O51C—C12C—C11C	110.11 (10)
C2A—C3A—H3A1	109.6	C13C—C12C—C11C	112.48 (8)
C3A—C4A—C5A	111.52 (9)	O51C—C12C—H12C1	108.9
C3A—C4A—H4A1	109.3	C13C—C12C—H12C1	108.9
C5A—C4A—H4A1	109.3	C11C—C12C—H12C1	108.9
C3A—C4A—H4A2	109.3	C12C—C13C—C18C	108.21 (9)
C5A—C4A—H4A2	109.3	C12C—C13C—C14C	108.09 (8)
H4A1—C4A—H4A2	108.0	C18C—C13C—C14C	112.95 (10)
C6A—C5A—C4A	112.20 (9)	C12C—C13C—C17C	117.34 (8)
C6A—C5A—C10A	111.29 (8)	C18C—C13C—C17C	110.47 (10)
C4A—C5A—C10A	112.67 (10)	C14C—C13C—C17C	99.68 (8)
C6A—C5A—H5A1	106.7	C8C—C14C—C15C	118.09 (9)
C4A—C5A—H5A1	106.7	C8C—C14C—C13C	113.34 (8)
C10A—C5A—H5A1	106.7	C15C—C14C—C13C	103.92 (8)
C7A—C6A—C5A	114.72 (8)	C8C—C14C—H14C1	107.0
C7A—C6A—H6A1	108.6	C15C—C14C—H14C1	107.0
C5A—C6A—H6A1	108.6	C13C—C14C—H14C1	107.0
C7A—C6A—H6A2	108.6	C14C—C15C—C16C	104.00 (10)
C5A—C6A—H6A2	108.6	C14C—C15C—H15C1	111.0
H6A1—C6A—H6A2	107.6	C16C—C15C—H15C1	111.0
O71A—C7A—C6A	110.02 (9)	C14C—C15C—H15C2	111.0
O71A—C7A—C8A	108.44 (9)	C16C—C15C—H15C2	111.0
C6A—C7A—C8A	113.09 (8)	H15C1—C15C—H15C2	109.0
O71A—C7A—H7A1	108.4	C15C—C16C—C17C	106.90 (9)
C6A—C7A—H7A1	108.4	C15C—C16C—H16C1	110.3
C8A—C7A—H7A1	108.4	C17C—C16C—H16C1	110.3
C7A—C8A—C14A	113.96 (7)	C15C—C16C—H16C2	110.3
C7A—C8A—C9A	113.31 (8)	C17C—C16C—H16C2	110.3
C14A—C8A—C9A	108.50 (7)	H16C1—C16C—H16C2	108.6
C7A—C8A—H8A1	106.9	C20C—C17C—C16C	112.51 (10)
C14A—C8A—H8A1	106.9	C20C—C17C—C13C	119.31 (8)
C9A—C8A—H8A1	106.9	C16C—C17C—C13C	102.97 (9)
C11A—C9A—C8A	110.50 (8)	C20C—C17C—H17C1	107.1
C11A—C9A—C10A	112.06 (7)	C16C—C17C—H17C1	107.1
C8A—C9A—C10A	112.28 (7)	C13C—C17C—H17C1	107.1
C11A—C9A—H9A1	107.2	C13C—C18C—H18C1	109.5
C8A—C9A—H9A1	107.2	C13C—C18C—H18C2	109.5
C10A—C9A—H9A1	107.2	H18C1—C18C—H18C2	109.5
C19A—C10A—C1A	106.58 (9)	C13C—C18C—H18C3	109.5
C19A—C10A—C5A	109.70 (10)	H18C1—C18C—H18C3	109.5
C1A—C10A—C5A	107.98 (8)	H18C2—C18C—H18C3	109.5
C19A—C10A—C9A	111.75 (8)	C10C—C19C—H19C1	109.5
C1A—C10A—C9A	112.36 (8)	C10C—C19C—H19C2	109.5
C5A—C10A—C9A	108.39 (7)	H19C1—C19C—H19C2	109.5
C12A—C11A—C9A	115.53 (7)	C10C—C19C—H19C3	109.5
C12A—C11A—H11A1	108.4	H19C1—C19C—H19C3	109.5
C9A—C11A—H11A1	108.4	H19C2—C19C—H19C3	109.5
C12A—C11A—H11A2	108.4	C21C—C20C—C17C	114.12 (11)
C9A—C11A—H11A2	108.4	C21C—C20C—C22C	110.26 (11)
H11A1—C11A—H11A2	107.5	C17C—C20C—C22C	110.58 (10)
O51A—C12A—C11A	110.15 (9)	C21C—C20C—H20C1	107.2
O51A—C12A—C13A	107.55 (8)	C17C—C20C—H20C1	107.2
C11A—C12A—C13A	112.33 (8)	C22C—C20C—H20C1	107.2
O51A—C12A—H12A1	108.9	C20C—C21C—H21C1	109.5
C11A—C12A—H12A1	108.9	C20C—C21C—H21C2	109.5
C13A—C12A—H12A1	108.9	H21C1—C21C—H21C2	109.5
C12A—C13A—C18A	108.91 (9)	C20C—C21C—H21C3	109.5
C12A—C13A—C14A	108.25 (7)	H21C1—C21C—H21C3	109.5
C18A—C13A—C14A	112.69 (10)	H21C2—C21C—H21C3	109.5
C12A—C13A—C17A	116.06 (8)	C23C—C22C—C20C	114.57 (11)
C18A—C13A—C17A	110.34 (8)	C23C—C22C—H22C1	108.6
C14A—C13A—C17A	100.45 (7)	C20C—C22C—H22C1	108.6
C8A—C14A—C15A	119.69 (9)	C23C—C22C—H22C2	108.6
C8A—C14A—C13A	112.85 (7)	C20C—C22C—H22C2	108.6
C15A—C14A—C13A	103.62 (8)	H22C1—C22C—H22C2	107.6
C8A—C14A—H14A1	106.6	C24C—C23C—C22C	111.51 (11)
C15A—C14A—H14A1	106.6	C24C—C23C—H23C1	109.3
C13A—C14A—H14A1	106.6	C22C—C23C—H23C1	109.3
C14A—C15A—C16A	103.80 (9)	C24C—C23C—H23C2	109.3
C14A—C15A—H15A1	111.0	C22C—C23C—H23C2	109.3
C16A—C15A—H15A1	111.0	H23C1—C23C—H23C2	108.0
C14A—C15A—H15A2	111.0	C23C—C24C—S25C	113.58 (10)
C16A—C15A—H15A2	111.0	C23C—C24C—H24C1	108.9
H15A1—C15A—H15A2	109.0	S25C—C24C—H24C1	108.9
C15A—C16A—C17A	107.11 (8)	C23C—C24C—H24C2	108.9
C15A—C16A—H16A1	110.3	S25C—C24C—H24C2	108.9
C17A—C16A—H16A1	110.3	H24C1—C24C—H24C2	107.7
C15A—C16A—H16A2	110.3	C24C—S25C—S25D	103.76 (6)
C17A—C16A—H16A2	110.3	C32C—O31C—C3C	118.77 (10)
H16A1—C16A—H16A2	108.5	O33C—C32C—O31C	126.05 (11)
C20A—C17A—C13A	119.07 (8)	O33C—C32C—C34C	122.97 (11)
C20A—C17A—C16A	112.51 (8)	O31C—C32C—C34C	110.96 (10)
C13A—C17A—C16A	102.90 (7)	C35C—C34C—C39C	120.08 (10)
C20A—C17A—H17A1	107.2	C35C—C34C—C32C	118.14 (10)
C13A—C17A—H17A1	107.2	C39C—C34C—C32C	121.69 (10)
C16A—C17A—H17A1	107.2	C36C—C35C—C34C	120.46 (11)
C13A—C18A—H18A1	109.5	C36C—C35C—H35C1	119.8
C13A—C18A—H18A2	109.5	C34C—C35C—H35C1	119.8
H18A1—C18A—H18A2	109.5	C37C—C36C—C35C	117.94 (10)
C13A—C18A—H18A3	109.5	C37C—C36C—H36C1	121.0
H18A1—C18A—H18A3	109.5	C35C—C36C—H36C1	121.0
H18A2—C18A—H18A3	109.5	C36C—C37C—C38C	123.07 (10)
C10A—C19A—H19A1	109.5	C36C—C37C—N40C	119.00 (10)
C10A—C19A—H19A2	109.5	C38C—C37C—N40C	117.93 (10)
H19A1—C19A—H19A2	109.5	C39C—C38C—C37C	118.13 (10)
C10A—C19A—H19A3	109.5	C39C—C38C—H38C1	120.9
H19A1—C19A—H19A3	109.5	C37C—C38C—H38C1	120.9
H19A2—C19A—H19A3	109.5	C38C—C39C—C34C	120.32 (10)
C21A—C20A—C17A	114.83 (9)	C38C—C39C—H39C1	119.8
C21A—C20A—C22A	109.53 (9)	C34C—C39C—H39C1	119.8
C17A—C20A—C22A	110.30 (9)	O41C—N40C—O42C	123.33 (11)
C21A—C20A—H20A1	107.3	O41C—N40C—C37C	118.29 (11)
C17A—C20A—H20A1	107.3	O42C—N40C—C37C	118.38 (10)
C22A—C20A—H20A1	107.3	C52C—O51C—C12C	116.53 (8)
C20A—C21A—H21A1	109.5	O53C—C52C—O51C	125.16 (10)
C20A—C21A—H21A2	109.5	O53C—C52C—C54C	122.36 (11)
H21A1—C21A—H21A2	109.5	O51C—C52C—C54C	112.48 (8)
C20A—C21A—H21A3	109.5	C59C—C54C—C55C	120.53 (9)
H21A1—C21A—H21A3	109.5	C59C—C54C—C52C	121.85 (11)
H21A2—C21A—H21A3	109.5	C55C—C54C—C52C	117.62 (9)
C23A—C22A—C20A	114.61 (8)	C56C—C55C—C54C	120.07 (9)
C23A—C22A—H22A1	108.6	C56C—C55C—H55C1	120.0
C20A—C22A—H22A1	108.6	C54C—C55C—H55C1	120.0
C23A—C22A—H22A2	108.6	C57C—C56C—C55C	118.32 (11)
C20A—C22A—H22A2	108.6	C57C—C56C—H56C1	120.8
H22A1—C22A—H22A2	107.6	C55C—C56C—H56C1	120.8
C24A—C23A—C22A	113.56 (9)	C58C—C57C—C56C	122.68 (9)
C24A—C23A—H23A1	108.9	C58C—C57C—N60C	118.48 (9)
C22A—C23A—H23A1	108.9	C56C—C57C—N60C	118.75 (11)
C24A—C23A—H23A2	108.9	C57C—C58C—C59C	118.68 (9)
C22A—C23A—H23A2	108.9	C57C—C58C—H58C1	120.7
H23A1—C23A—H23A2	107.7	C59C—C58C—H58C1	120.7
C23A—C24A—S25A	115.45 (10)	C58C—C59C—C54C	119.62 (11)
C23A—C24A—H24A1	108.4	C58C—C59C—H59C1	120.2
S25A—C24A—H24A1	108.4	C54C—C59C—H59C1	120.2
C23A—C24A—H24A2	108.4	O61C—N60C—O62C	124.42 (10)
S25A—C24A—H24A2	108.4	O61C—N60C—C57C	118.31 (10)
H24A1—C24A—H24A2	107.5	O62C—N60C—C57C	117.22 (11)
C24A—S25A—S25B	103.11 (6)	C72C—O71C—C7C	117.91 (10)
C32A—O31A—C3A	116.21 (9)	O73C—C72C—O71C	125.30 (10)
O33A—C32A—O31A	124.66 (11)	O73C—C72C—C74C	123.04 (10)
O33A—C32A—C34A	122.71 (11)	O71C—C72C—C74C	111.67 (10)
O31A—C32A—C34A	112.63 (10)	C75C—C74C—C79C	120.18 (10)
C35A—C34A—C39A	120.46 (10)	C75C—C74C—C72C	118.09 (10)
C35A—C34A—C32A	117.08 (10)	C79C—C74C—C72C	121.73 (9)
C39A—C34A—C32A	122.46 (10)	C76C—C75C—C74C	120.65 (11)
C36A—C35A—C34A	120.46 (11)	C76C—C75C—H75C1	119.7
C36A—C35A—H35A1	119.8	C74C—C75C—H75C1	119.7
C34A—C35A—H35A1	119.8	C77C—C76C—C75C	117.76 (10)
C37A—C36A—C35A	117.93 (11)	C77C—C76C—H76C1	121.1
C37A—C36A—H36A1	121.0	C75C—C76C—H76C1	121.1
C35A—C36A—H36A1	121.0	C78C—C77C—C76C	122.96 (9)
C36A—C37A—C38A	123.20 (11)	C78C—C77C—N80C	118.09 (11)
C36A—C37A—N40A	118.18 (11)	C76C—C77C—N80C	118.89 (10)
C38A—C37A—N40A	118.62 (11)	C77C—C78C—C79C	118.66 (11)
C37A—C38A—C39A	118.24 (11)	C77C—C78C—H78C1	120.7
C37A—C38A—H38A1	120.9	C79C—C78C—H78C1	120.7
C39A—C38A—H38A1	120.9	C78C—C79C—C74C	119.76 (10)
C38A—C39A—C34A	119.70 (11)	C78C—C79C—H79C1	120.1
C38A—C39A—H39A1	120.2	C74C—C79C—H79C1	120.1
C34A—C39A—H39A1	120.2	O81C—N80C—O82C	124.22 (11)
O41A—N40A—O42A	124.46 (12)	O81C—N80C—C77C	117.94 (11)
O41A—N40A—C37A	118.10 (12)	O82C—N80C—C77C	117.83 (9)
O42A—N40A—C37A	117.44 (11)	C2D—C1D—C10D	114.76 (9)
C52A—O51A—C12A	116.93 (9)	C2D—C1D—H1D1	108.6
O53A—C52A—O51A	124.81 (11)	C10D—C1D—H1D1	108.6
O53A—C52A—C54A	123.50 (11)	C2D—C1D—H1D2	108.6
O51A—C52A—C54A	111.68 (9)	C10D—C1D—H1D2	108.6
C55A—C54A—C59A	119.89 (11)	H1D1—C1D—H1D2	107.6
C55A—C54A—C52A	118.49 (10)	C3D—C2D—C1D	108.61 (11)
C59A—C54A—C52A	121.62 (10)	C3D—C2D—H2D1	110.0
C56A—C55A—C54A	120.32 (13)	C1D—C2D—H2D1	110.0
C56A—C55A—H55A1	119.8	C3D—C2D—H2D2	110.0
C54A—C55A—H55A1	119.8	C1D—C2D—H2D2	110.0
C57A—C56A—C55A	118.62 (12)	H2D1—C2D—H2D2	108.3
C57A—C56A—H56A1	120.7	O31D—C3D—C2D	110.13 (10)
C55A—C56A—H56A1	120.7	O31D—C3D—C4D	105.16 (10)
C56A—C57A—C58A	122.31 (12)	C2D—C3D—C4D	111.26 (9)
C56A—C57A—N60A	119.81 (13)	O31D—C3D—H3D1	110.1
C58A—C57A—N60A	117.81 (13)	C2D—C3D—H3D1	110.1
C57A—C58A—C59A	118.44 (12)	C4D—C3D—H3D1	110.1
C57A—C58A—H58A1	120.8	C3D—C4D—C5D	110.91 (10)
C59A—C58A—H58A1	120.8	C3D—C4D—H4D1	109.5
C58A—C59A—C54A	120.20 (11)	C5D—C4D—H4D1	109.5
C58A—C59A—H59A1	119.9	C3D—C4D—H4D2	109.5
C54A—C59A—H59A1	119.9	C5D—C4D—H4D2	109.5
C62A—N60A—O61A	125.17 (14)	H4D1—C4D—H4D2	108.0
C62A—N60A—C57A	117.71 (13)	C6D—C5D—C4D	111.20 (10)
O61A—N60A—C57A	117.12 (14)	C6D—C5D—C10D	112.36 (8)
C72A—O71A—C7A	114.66 (9)	C4D—C5D—C10D	113.20 (10)
O73A—C72A—O71A	124.60 (10)	C6D—C5D—H5D1	106.5
O73A—C72A—C74A	122.69 (9)	C4D—C5D—H5D1	106.5
O71A—C72A—C74A	112.70 (10)	C10D—C5D—H5D1	106.5
C79A—C74A—C75A	120.42 (9)	C7D—C6D—C5D	114.32 (8)
C79A—C74A—C72A	122.62 (9)	C7D—C6D—H6D1	108.7
C75A—C74A—C72A	116.92 (10)	C5D—C6D—H6D1	108.7
C76A—C75A—C74A	120.83 (11)	C7D—C6D—H6D2	108.7
C76A—C75A—H75A1	119.6	C5D—C6D—H6D2	108.7
C74A—C75A—H75A1	119.6	H6D1—C6D—H6D2	107.6
C75A—C76A—C77A	117.60 (10)	O71D—C7D—C6D	110.53 (10)
C75A—C76A—H76A1	121.2	O71D—C7D—C8D	106.62 (8)
C77A—C76A—H76A1	121.2	C6D—C7D—C8D	112.35 (9)
C78A—C77A—C76A	122.86 (10)	O71D—C7D—H7D1	109.1
C78A—C77A—N80A	118.28 (11)	C6D—C7D—H7D1	109.1
C76A—C77A—N80A	118.83 (10)	C8D—C7D—H7D1	109.1
C77A—C78A—C79A	119.02 (11)	C14D—C8D—C7D	112.02 (8)
C77A—C78A—H78A1	120.5	C14D—C8D—C9D	110.17 (7)
C79A—C78A—H78A1	120.5	C7D—C8D—C9D	112.17 (8)
C74A—C79A—C78A	119.16 (9)	C14D—C8D—H8D1	107.4
C74A—C79A—H79A1	120.4	C7D—C8D—H8D1	107.4
C78A—C79A—H79A1	120.4	C9D—C8D—H8D1	107.4
O81A—N80A—C82A	124.05 (11)	C11D—C9D—C8D	110.96 (8)
O81A—N80A—C77A	118.34 (12)	C11D—C9D—C10D	112.94 (8)
C82A—N80A—C77A	117.60 (11)	C8D—C9D—C10D	110.77 (7)
C2B—C1B—C10B	114.75 (8)	C11D—C9D—H9D1	107.3
C2B—C1B—H1B1	108.6	C8D—C9D—H9D1	107.3
C10B—C1B—H1B1	108.6	C10D—C9D—H9D1	107.3
C2B—C1B—H1B2	108.6	C19D—C10D—C1D	105.86 (10)
C10B—C1B—H1B2	108.6	C19D—C10D—C5D	108.74 (9)
H1B1—C1B—H1B2	107.6	C1D—C10D—C5D	108.08 (8)
C3B—C2B—C1B	108.82 (11)	C19D—C10D—C9D	111.54 (9)
C3B—C2B—H2B1	109.9	C1D—C10D—C9D	113.05 (8)
C1B—C2B—H2B1	109.9	C5D—C10D—C9D	109.41 (8)
C3B—C2B—H2B2	109.9	C12D—C11D—C9D	115.33 (7)
C1B—C2B—H2B2	109.9	C12D—C11D—H11D1	108.4
H2B1—C2B—H2B2	108.3	C9D—C11D—H11D1	108.4
O31B—C3B—C2B	110.92 (10)	C12D—C11D—H11D2	108.4
O31B—C3B—C4B	104.93 (9)	C9D—C11D—H11D2	108.4
C2B—C3B—C4B	111.29 (8)	H11D1—C11D—H11D2	107.5
O31B—C3B—H3B1	109.9	O51D—C12D—C11D	108.50 (9)
C2B—C3B—H3B1	109.9	O51D—C12D—C13D	107.75 (8)
C4B—C3B—H3B1	109.9	C11D—C12D—C13D	111.87 (7)
C3B—C4B—C5B	112.08 (9)	O51D—C12D—H12D1	109.6
C3B—C4B—H4B1	109.2	C11D—C12D—H12D1	109.6
C5B—C4B—H4B1	109.2	C13D—C12D—H12D1	109.6
C3B—C4B—H4B2	109.2	C12D—C13D—C18D	108.29 (8)
C5B—C4B—H4B2	109.2	C12D—C13D—C14D	107.46 (7)
H4B1—C4B—H4B2	107.9	C18D—C13D—C14D	112.60 (8)
C6B—C5B—C4B	111.52 (9)	C12D—C13D—C17D	118.07 (7)
C6B—C5B—C10B	111.87 (8)	C18D—C13D—C17D	109.98 (9)
C4B—C5B—C10B	112.70 (10)	C14D—C13D—C17D	100.32 (7)
C6B—C5B—H5B1	106.8	C8D—C14D—C15D	117.48 (8)
C4B—C5B—H5B1	106.8	C8D—C14D—C13D	113.11 (8)
C10B—C5B—H5B1	106.8	C15D—C14D—C13D	104.16 (7)
C7B—C6B—C5B	114.58 (9)	C8D—C14D—H14D1	107.2
C7B—C6B—H6B1	108.6	C15D—C14D—H14D1	107.2
C5B—C6B—H6B1	108.6	C13D—C14D—H14D1	107.2
C7B—C6B—H6B2	108.6	C14D—C15D—C16D	104.17 (8)
C5B—C6B—H6B2	108.6	C14D—C15D—H15D1	110.9
H6B1—C6B—H6B2	107.6	C16D—C15D—H15D1	110.9
O71B—C7B—C6B	110.40 (8)	C14D—C15D—H15D2	110.9
O71B—C7B—C8B	106.52 (9)	C16D—C15D—H15D2	110.9
C6B—C7B—C8B	113.67 (7)	H15D1—C15D—H15D2	108.9
O71B—C7B—H7B1	108.7	C15D—C16D—C17D	107.12 (8)
C6B—C7B—H7B1	108.7	C15D—C16D—H16D1	110.3
C8B—C7B—H7B1	108.7	C17D—C16D—H16D1	110.3
C14B—C8B—C7B	111.68 (7)	C15D—C16D—H16D2	110.3
C14B—C8B—C9B	110.21 (9)	C17D—C16D—H16D2	110.3
C7B—C8B—C9B	111.02 (8)	H16D1—C16D—H16D2	108.5
C14B—C8B—H8B1	107.9	C20D—C17D—C16D	111.36 (8)
C7B—C8B—H8B1	107.9	C20D—C17D—C13D	118.73 (7)
C9B—C8B—H8B1	107.9	C16D—C17D—C13D	102.82 (7)
C11B—C9B—C8B	111.59 (8)	C20D—C17D—H17D1	107.8
C11B—C9B—C10B	113.28 (7)	C16D—C17D—H17D1	107.8
C8B—C9B—C10B	110.87 (9)	C13D—C17D—H17D1	107.8
C11B—C9B—H9B1	106.9	C13D—C18D—H18D1	109.5
C8B—C9B—H9B1	106.9	C13D—C18D—H18D2	109.5
C10B—C9B—H9B1	106.9	H18D1—C18D—H18D2	109.5
C1B—C10B—C19B	105.93 (9)	C13D—C18D—H18D3	109.5
C1B—C10B—C5B	107.81 (8)	H18D1—C18D—H18D3	109.5
C19B—C10B—C5B	109.74 (10)	H18D2—C18D—H18D3	109.5
C1B—C10B—C9B	112.71 (9)	C10D—C19D—H19D1	109.5
C19B—C10B—C9B	111.86 (8)	C10D—C19D—H19D2	109.5
C5B—C10B—C9B	108.68 (7)	H19D1—C19D—H19D2	109.5
C12B—C11B—C9B	114.90 (7)	C10D—C19D—H19D3	109.5
C12B—C11B—H11B1	108.5	H19D1—C19D—H19D3	109.5
C9B—C11B—H11B1	108.5	H19D2—C19D—H19D3	109.5
C12B—C11B—H11B2	108.5	C21D—C20D—C22D	110.49 (9)
C9B—C11B—H11B2	108.5	C21D—C20D—C17D	113.11 (9)
H11B1—C11B—H11B2	107.5	C22D—C20D—C17D	111.75 (8)
O51B—C12B—C11B	109.49 (8)	C21D—C20D—H20D1	107.0
O51B—C12B—C13B	107.72 (8)	C22D—C20D—H20D1	107.0
C11B—C12B—C13B	112.41 (8)	C17D—C20D—H20D1	107.0
O51B—C12B—H12B1	109.1	C20D—C21D—H21D1	109.5
C11B—C12B—H12B1	109.1	C20D—C21D—H21D2	109.5
C13B—C12B—H12B1	109.1	H21D1—C21D—H21D2	109.5
C12B—C13B—C18B	107.87 (8)	C20D—C21D—H21D3	109.5
C12B—C13B—C14B	108.11 (8)	H21D1—C21D—H21D3	109.5
C18B—C13B—C14B	113.08 (10)	H21D2—C21D—H21D3	109.5
C12B—C13B—C17B	118.04 (8)	C23D—C22D—C20D	115.41 (11)
C18B—C13B—C17B	110.11 (7)	C23D—C22D—H22D1	108.4
C14B—C13B—C17B	99.58 (7)	C20D—C22D—H22D1	108.4
C8B—C14B—C15B	118.19 (9)	C23D—C22D—H22D2	108.4
C8B—C14B—C13B	113.85 (7)	C20D—C22D—H22D2	108.4
C15B—C14B—C13B	104.19 (8)	H22D1—C22D—H22D2	107.5
C8B—C14B—H14B1	106.6	C24D—C23D—C22D	110.82 (13)
C15B—C14B—H14B1	106.6	C24D—C23D—H23D1	109.5
C13B—C14B—H14B1	106.6	C22D—C23D—H23D1	109.5
C14B—C15B—C16B	103.75 (10)	C24D—C23D—H23D2	109.5
C14B—C15B—H15B1	111.0	C22D—C23D—H23D2	109.5
C16B—C15B—H15B1	111.0	H23D1—C23D—H23D2	108.1
C14B—C15B—H15B2	111.0	C23D—C24D—S25D	113.75 (13)
C16B—C15B—H15B2	111.0	C23D—C24D—H24D1	108.8
H15B1—C15B—H15B2	109.0	S25D—C24D—H24D1	108.8
C15B—C16B—C17B	107.02 (8)	C23D—C24D—H24D2	108.8
C15B—C16B—H16B1	110.3	S25D—C24D—H24D2	108.8
C17B—C16B—H16B1	110.3	H24D1—C24D—H24D2	107.7
C15B—C16B—H16B2	110.3	C24D—S25D—S25C	103.56 (5)
C17B—C16B—H16B2	110.3	C32D—O31D—C3D	117.46 (10)
H16B1—C16B—H16B2	108.6	O33D—C32D—O31D	125.74 (11)
C20B—C17B—C16B	112.37 (8)	O33D—C32D—C34D	123.44 (10)
C20B—C17B—C13B	119.07 (8)	O31D—C32D—C34D	110.82 (11)
C16B—C17B—C13B	102.62 (7)	C35D—C34D—C39D	120.35 (9)
C20B—C17B—H17B1	107.4	C35D—C34D—C32D	117.91 (10)
C16B—C17B—H17B1	107.4	C39D—C34D—C32D	121.74 (9)
C13B—C17B—H17B1	107.4	C36D—C35D—C34D	120.26 (11)
C13B—C18B—H18B1	109.5	C36D—C35D—H35D1	119.9
C13B—C18B—H18B2	109.5	C34D—C35D—H35D1	119.9
H18B1—C18B—H18B2	109.5	C37D—C36D—C35D	117.98 (10)
C13B—C18B—H18B3	109.5	C37D—C36D—H36D1	121.0
H18B1—C18B—H18B3	109.5	C35D—C36D—H36D1	121.0
H18B2—C18B—H18B3	109.5	C36D—C37D—C38D	123.19 (9)
C10B—C19B—H19B1	109.5	C36D—C37D—N40D	118.67 (9)
C10B—C19B—H19B2	109.5	C38D—C37D—N40D	118.14 (10)
H19B1—C19B—H19B2	109.5	C39D—C38D—C37D	118.04 (10)
C10B—C19B—H19B3	109.5	C39D—C38D—H38D1	121.0
H19B1—C19B—H19B3	109.5	C37D—C38D—H38D1	121.0
H19B2—C19B—H19B3	109.5	C38D—C39D—C34D	120.16 (9)
C21B—C20B—C17B	112.43 (8)	C38D—C39D—H39D1	119.9
C21B—C20B—C22B	110.36 (9)	C34D—C39D—H39D1	119.9
C17B—C20B—C22B	111.56 (9)	O41D—N40D—O42D	124.15 (11)
C21B—C20B—H20B1	107.4	O41D—N40D—C37D	117.98 (11)
C17B—C20B—H20B1	107.4	O42D—N40D—C37D	117.86 (10)
C22B—C20B—H20B1	107.4	C52D—O51D—C12D	116.97 (8)
C20B—C21B—H21B1	109.5	O53D—C52D—O51D	124.78 (9)
C20B—C21B—H21B2	109.5	O53D—C52D—C54D	123.15 (10)
H21B1—C21B—H21B2	109.5	O51D—C52D—C54D	112.06 (8)
C20B—C21B—H21B3	109.5	C55D—C54D—C59D	120.56 (8)
H21B1—C21B—H21B3	109.5	C55D—C54D—C52D	117.91 (8)
H21B2—C21B—H21B3	109.5	C59D—C54D—C52D	121.52 (9)
C23B—C22B—C20B	114.70 (8)	C56D—C55D—C54D	120.56 (9)
C23B—C22B—H22B1	108.6	C56D—C55D—H55D1	119.7
C20B—C22B—H22B1	108.6	C54D—C55D—H55D1	119.7
C23B—C22B—H22B2	108.6	C57D—C56D—C55D	117.66 (10)
C20B—C22B—H22B2	108.6	C57D—C56D—H56D1	121.2
H22B1—C22B—H22B2	107.6	C55D—C56D—H56D1	121.2
C24B—C23B—C22B	113.25 (9)	C56D—C57D—C58D	123.04 (9)
C24B—C23B—H23B1	108.9	C56D—C57D—N60D	119.27 (10)
C22B—C23B—H23B1	108.9	C58D—C57D—N60D	117.69 (9)
C24B—C23B—H23B2	108.9	C59D—C58D—C57D	118.85 (9)
C22B—C23B—H23B2	108.9	C59D—C58D—H58D1	120.6
H23B1—C23B—H23B2	107.7	C57D—C58D—H58D1	120.6
C23B—C24B—S25B	115.74 (9)	C58D—C59D—C54D	119.32 (10)
C23B—C24B—H24B1	108.3	C58D—C59D—H59D1	120.3
S25B—C24B—H24B1	108.3	C54D—C59D—H59D1	120.3
C23B—C24B—H24B2	108.3	O61D—N60D—O62D	123.99 (10)
S25B—C24B—H24B2	108.3	O61D—N60D—C57D	118.30 (9)
H24B1—C24B—H24B2	107.4	O62D—N60D—C57D	117.70 (10)
C24B—S25B—S25A	103.22 (5)	C72D—O71D—C7D	118.04 (9)
C32B—O31B—C3B	117.32 (9)	O73D—C72D—O71D	125.16 (10)
O33B—C32B—O31B	125.14 (11)	O73D—C72D—C74D	123.65 (10)
O33B—C32B—C34B	123.48 (10)	O71D—C72D—C74D	111.17 (10)
O31B—C32B—C34B	111.38 (10)	C75D—C74D—C79D	120.30 (10)
C35B—C34B—C39B	120.07 (10)	C75D—C74D—C72D	118.21 (10)
C35B—C34B—C32B	118.28 (10)	C79D—C74D—C72D	121.48 (9)
C39B—C34B—C32B	121.64 (10)	C74D—C75D—C76D	120.31 (11)
C36B—C35B—C34B	120.50 (10)	C74D—C75D—H75D1	119.8
C36B—C35B—H35B1	119.7	C76D—C75D—H75D1	119.8
C34B—C35B—H35B1	119.7	C77D—C76D—C75D	117.91 (10)
C37B—C36B—C35B	118.06 (11)	C77D—C76D—H76D1	121.0
C37B—C36B—H36B1	121.0	C75D—C76D—H76D1	121.0
C35B—C36B—H36B1	121.0	C76D—C77D—C78D	123.08 (10)
C36B—C37B—C38B	123.15 (11)	C76D—C77D—N80D	118.69 (10)
C36B—C37B—N40B	118.84 (11)	C78D—C77D—N80D	118.22 (12)
C38B—C37B—N40B	118.01 (11)	C77D—C78D—C79D	118.46 (11)
C37B—C38B—C39B	118.08 (11)	C77D—C78D—H78D1	120.8
C37B—C38B—H38B1	121.0	C79D—C78D—H78D1	120.8
C39B—C38B—H38B1	121.0	C78D—C79D—C74D	119.92 (10)
C38B—C39B—C34B	120.14 (11)	C78D—C79D—H79D1	120.0
C38B—C39B—H39B1	119.9	C74D—C79D—H79D1	120.0
C34B—C39B—H39B1	119.9	O81D—N80D—O82D	123.52 (11)
O41B—N40B—O42B	124.11 (13)	O81D—N80D—C77D	118.07 (13)
O41B—N40B—C37B	118.62 (12)	O82D—N80D—C77D	118.40 (11)
O42B—N40B—C37B	117.26 (13)	C102—C101—H101A	109.5
C52B—O51B—C12B	116.73 (8)	C102—C101—H101B	109.5
O53B—C52B—O51B	124.68 (9)	H101A—C101—H101B	109.5
O53B—C52B—C54B	123.20 (11)	C102—C101—H101C	109.5
O51B—C52B—C54B	112.10 (8)	H101A—C101—H101C	109.5
C59B—C54B—C55B	120.46 (9)	H101B—C101—H101C	109.5
C59B—C54B—C52B	121.58 (10)	O103—C102—O104	123.76 (13)
C55B—C54B—C52B	117.94 (9)	O103—C102—C101	124.85 (11)
C56B—C55B—C54B	119.89 (10)	O104—C102—C101	111.36 (11)
C56B—C55B—H55B1	120.1	C102—O104—C105	117.40 (10)
C54B—C55B—H55B1	120.1	O104—C105—C106	108.00 (11)
C57B—C56B—C55B	118.29 (11)	O104—C105—H105A	110.1
C57B—C56B—H56B1	120.9	C106—C105—H105A	110.1
C55B—C56B—H56B1	120.9	O104—C105—H105B	110.1
C56B—C57B—C58B	123.01 (9)	C106—C105—H105B	110.1
C56B—C57B—N60B	119.28 (11)	H105A—C105—H105B	108.4
C58B—C57B—N60B	117.69 (10)	C105—C106—H106A	109.5
C57B—C58B—C59B	118.40 (9)	C105—C106—H106B	109.5
C57B—C58B—H58B1	120.8	H106A—C106—H106B	109.5
C59B—C58B—H58B1	120.8	C105—C106—H106C	109.5
C54B—C59B—C58B	119.88 (10)	H106A—C106—H106C	109.5
C54B—C59B—H59B1	120.1	H106B—C106—H106C	109.5
C58B—C59B—H59B1	120.1	C202—C201—H201A	109.5
O61B—N60B—O62B	124.39 (11)	C202—C201—H201B	109.5
O61B—N60B—C57B	117.81 (12)	H201A—C201—H201B	109.5
O62B—N60B—C57B	117.79 (12)	C202—C201—H201C	109.5
C72B—O71B—C7B	116.59 (9)	H201A—C201—H201C	109.5
O73B—C72B—O71B	124.85 (10)	H201B—C201—H201C	109.5
O73B—C72B—C74B	123.41 (9)	O203—C202—O204	120.64 (15)
O71B—C72B—C74B	111.75 (10)	O203—C202—C201	121.00 (15)
C75B—C74B—C79B	120.57 (9)	O204—C202—C201	118.36 (15)
C75B—C74B—C72B	117.38 (10)	C202—O204—C205	111.79 (14)
C79B—C74B—C72B	122.06 (9)	O204—C205—C206	107.92 (15)
C76B—C75B—C74B	120.64 (11)	O204—C205—H205A	110.1
C76B—C75B—H75B1	119.7	C206—C205—H205A	110.1
C74B—C75B—H75B1	119.7	O204—C205—H205B	110.1
C77B—C76B—C75B	117.86 (10)	C206—C205—H205B	110.1
C77B—C76B—H76B1	121.1	H205A—C205—H205B	108.4
C75B—C76B—H76B1	121.1	C205—C206—H206A	109.5
C76B—C77B—C78B	122.93 (9)	C205—C206—H206B	109.5
C76B—C77B—N80B	118.70 (10)	H206A—C206—H206B	109.5
C78B—C77B—N80B	118.37 (11)	C205—C206—H206C	109.5
C77B—C78B—C79B	118.61 (11)	H206A—C206—H206C	109.5
C77B—C78B—H78B1	120.7	H206B—C206—H206C	109.5
C79B—C78B—H78B1	120.7	C212—C211—H211A	109.5
C78B—C79B—C74B	119.40 (9)	C212—C211—H211B	109.5
C78B—C79B—H79B1	120.3	H211A—C211—H211B	109.5
C74B—C79B—H79B1	120.3	C212—C211—H211C	109.5
O81B—N80B—O82B	124.27 (11)	H211A—C211—H211C	109.5
O81B—N80B—C77B	117.80 (12)	H211B—C211—H211C	109.5
O82B—N80B—C77B	117.93 (10)	O213—C212—O214	120.29 (15)
C2C—C1C—C10C	114.71 (10)	O213—C212—C211	121.75 (15)
C2C—C1C—H1C1	108.6	O214—C212—C211	117.96 (14)
C10C—C1C—H1C1	108.6	C212—O214—C215	109.49 (17)
C2C—C1C—H1C2	108.6	O214—C215—C216	109.50 (16)
C10C—C1C—H1C2	108.6	O214—C215—H215A	109.8
H1C1—C1C—H1C2	107.6	C216—C215—H215A	109.8
C3C—C2C—C1C	109.40 (11)	O214—C215—H215B	109.8
C3C—C2C—H2C1	109.8	C216—C215—H215B	109.8
C1C—C2C—H2C1	109.8	H215A—C215—H215B	108.2
C3C—C2C—H2C2	109.8	C215—C216—H216A	109.5
C1C—C2C—H2C2	109.8	C215—C216—H216B	109.5
H2C1—C2C—H2C2	108.2	H216A—C216—H216B	109.5
O31C—C3C—C4C	105.41 (11)	C215—C216—H216C	109.5
O31C—C3C—C2C	108.22 (10)	H216A—C216—H216C	109.5
C4C—C3C—C2C	111.52 (9)	H216B—C216—H216C	109.5
O31C—C3C—H3C1	110.5	C302—C301—H301A	109.5
C4C—C3C—H3C1	110.5	C302—C301—H301B	109.5
C2C—C3C—H3C1	110.5	H301A—C301—H301B	109.5
C3C—C4C—C5C	111.97 (11)	C302—C301—H301C	109.5
C3C—C4C—H4C1	109.2	H301A—C301—H301C	109.5
C5C—C4C—H4C1	109.2	H301B—C301—H301C	109.5
C3C—C4C—H4C2	109.2	C301—C302—C303	111.09 (16)
C5C—C4C—H4C2	109.2	C301—C302—H302A	109.4
H4C1—C4C—H4C2	107.9	C303—C302—H302A	109.4
C4C—C5C—C6C	111.13 (10)	C301—C302—H302B	109.4
C4C—C5C—C10C	112.97 (10)	C303—C302—H302B	109.4
C6C—C5C—C10C	112.13 (9)	H302A—C302—H302B	108.0
C4C—C5C—H5C1	106.7	C304—C303—C302	110.30 (15)
C6C—C5C—H5C1	106.7	C304—C303—H303A	109.6
C10C—C5C—H5C1	106.7	C302—C303—H303A	109.6
C7C—C6C—C5C	114.46 (8)	C304—C303—H303B	109.6
C7C—C6C—H6C1	108.6	C302—C303—H303B	109.6
C5C—C6C—H6C1	108.6	H303A—C303—H303B	108.1
C7C—C6C—H6C2	108.6	C305—C304—C303	111.70 (15)
C5C—C6C—H6C2	108.6	C305—C304—H304A	109.3
H6C1—C6C—H6C2	107.6	C303—C304—H304A	109.3
O71C—C7C—C6C	108.99 (10)	C305—C304—H304B	109.3
O71C—C7C—C8C	107.73 (9)	C303—C304—H304B	109.3
C6C—C7C—C8C	112.62 (9)	H304A—C304—H304B	107.9
O71C—C7C—H7C1	109.1	C304—C305—C306	110.43 (16)
C6C—C7C—H7C1	109.1	C304—C305—H305A	109.6
C8C—C7C—H7C1	109.1	C306—C305—H305A	109.6
C14C—C8C—C7C	112.19 (8)	C304—C305—H305B	109.6
C14C—C8C—C9C	110.02 (8)	C306—C305—H305B	109.6
C7C—C8C—C9C	111.69 (8)	H305A—C305—H305B	108.1
C14C—C8C—H8C1	107.6	C305—C306—H306A	109.5
C7C—C8C—H8C1	107.6	C305—C306—H306B	109.5
C9C—C8C—H8C1	107.6	H306A—C306—H306B	109.5
C11C—C9C—C8C	111.42 (8)	C305—C306—H306C	109.5
C11C—C9C—C10C	112.57 (8)	H306A—C306—H306C	109.5
C8C—C9C—C10C	110.56 (8)	H306B—C306—H306C	109.5
			
C10A—C1A—C2A—C3A	57.65 (11)	C3C—C4C—C5C—C10C	−54.51 (12)
C1A—C2A—C3A—O31A	−173.88 (8)	C4C—C5C—C6C—C7C	77.22 (13)
C1A—C2A—C3A—C4A	−56.56 (12)	C10C—C5C—C6C—C7C	−50.29 (12)
O31A—C3A—C4A—C5A	176.41 (7)	C5C—C6C—C7C—O71C	−71.49 (12)
C2A—C3A—C4A—C5A	56.01 (12)	C5C—C6C—C7C—C8C	48.00 (12)
C3A—C4A—C5A—C6A	178.84 (7)	O71C—C7C—C8C—C14C	−55.44 (11)
C3A—C4A—C5A—C10A	−54.62 (11)	C6C—C7C—C8C—C14C	−175.67 (8)
C4A—C5A—C6A—C7A	73.51 (11)	O71C—C7C—C8C—C9C	68.63 (11)
C10A—C5A—C6A—C7A	−53.77 (10)	C6C—C7C—C8C—C9C	−51.60 (10)
C5A—C6A—C7A—O71A	−74.46 (11)	C14C—C8C—C9C—C11C	−50.64 (9)
C5A—C6A—C7A—C8A	46.97 (11)	C7C—C8C—C9C—C11C	−175.92 (7)
O71A—C7A—C8A—C14A	−48.01 (11)	C14C—C8C—C9C—C10C	−176.62 (7)
C6A—C7A—C8A—C14A	−170.33 (8)	C7C—C8C—C9C—C10C	58.10 (10)
O71A—C7A—C8A—C9A	76.70 (11)	C2C—C1C—C10C—C19C	−171.86 (9)
C6A—C7A—C8A—C9A	−45.62 (10)	C2C—C1C—C10C—C5C	−54.48 (12)
C7A—C8A—C9A—C11A	178.17 (7)	C2C—C1C—C10C—C9C	66.04 (13)
C14A—C8A—C9A—C11A	−54.22 (9)	C4C—C5C—C10C—C19C	166.43 (9)
C7A—C8A—C9A—C10A	52.26 (9)	C6C—C5C—C10C—C19C	−67.06 (12)
C14A—C8A—C9A—C10A	179.88 (7)	C4C—C5C—C10C—C1C	51.69 (12)
C2A—C1A—C10A—C19A	−172.83 (8)	C6C—C5C—C10C—C1C	178.20 (8)
C2A—C1A—C10A—C5A	−55.03 (11)	C4C—C5C—C10C—C9C	−71.64 (11)
C2A—C1A—C10A—C9A	64.46 (11)	C6C—C5C—C10C—C9C	54.87 (10)
C6A—C5A—C10A—C19A	−64.80 (11)	C11C—C9C—C10C—C19C	−63.26 (12)
C4A—C5A—C10A—C19A	168.17 (8)	C8C—C9C—C10C—C19C	62.07 (11)
C6A—C5A—C10A—C1A	179.43 (7)	C11C—C9C—C10C—C1C	55.83 (11)
C4A—C5A—C10A—C1A	52.40 (11)	C8C—C9C—C10C—C1C	−178.83 (8)
C6A—C5A—C10A—C9A	57.46 (10)	C11C—C9C—C10C—C5C	175.59 (8)
C4A—C5A—C10A—C9A	−69.57 (11)	C8C—C9C—C10C—C5C	−59.07 (10)
C11A—C9A—C10A—C19A	−61.39 (12)	C8C—C9C—C11C—C12C	46.62 (11)
C8A—C9A—C10A—C19A	63.66 (11)	C10C—C9C—C11C—C12C	171.49 (8)
C11A—C9A—C10A—C1A	58.36 (10)	C9C—C11C—C12C—O51C	71.33 (12)
C8A—C9A—C10A—C1A	−176.59 (7)	C9C—C11C—C12C—C13C	−48.59 (11)
C11A—C9A—C10A—C5A	177.61 (8)	O51C—C12C—C13C—C18C	168.51 (8)
C8A—C9A—C10A—C5A	−57.34 (10)	C11C—C12C—C13C—C18C	−70.09 (12)
C8A—C9A—C11A—C12A	50.64 (11)	O51C—C12C—C13C—C14C	−68.86 (9)
C10A—C9A—C11A—C12A	176.67 (8)	C11C—C12C—C13C—C14C	52.54 (10)
C9A—C11A—C12A—O51A	69.58 (12)	O51C—C12C—C13C—C17C	42.74 (11)
C9A—C11A—C12A—C13A	−50.28 (11)	C11C—C12C—C13C—C17C	164.14 (8)
O51A—C12A—C13A—C18A	167.92 (7)	C7C—C8C—C14C—C15C	−53.14 (11)
C11A—C12A—C13A—C18A	−70.71 (10)	C9C—C8C—C14C—C15C	−178.14 (8)
O51A—C12A—C13A—C14A	−69.24 (10)	C7C—C8C—C14C—C13C	−174.97 (8)
C11A—C12A—C13A—C14A	52.13 (9)	C9C—C8C—C14C—C13C	60.04 (10)
O51A—C12A—C13A—C17A	42.72 (10)	C12C—C13C—C14C—C8C	−60.47 (10)
C11A—C12A—C13A—C17A	164.09 (7)	C18C—C13C—C14C—C8C	59.22 (12)
C7A—C8A—C14A—C15A	−48.87 (11)	C17C—C13C—C14C—C8C	176.44 (8)
C9A—C8A—C14A—C15A	−176.11 (7)	C12C—C13C—C14C—C15C	170.09 (8)
C7A—C8A—C14A—C13A	−171.17 (7)	C18C—C13C—C14C—C15C	−70.22 (12)
C9A—C8A—C14A—C13A	61.58 (9)	C17C—C13C—C14C—C15C	47.00 (9)
C12A—C13A—C14A—C8A	−60.16 (10)	C8C—C14C—C15C—C16C	−159.79 (8)
C18A—C13A—C14A—C8A	60.35 (11)	C13C—C14C—C15C—C16C	−33.28 (10)
C17A—C13A—C14A—C8A	177.75 (7)	C14C—C15C—C16C—C17C	6.28 (11)
C12A—C13A—C14A—C15A	168.91 (7)	C15C—C16C—C17C—C20C	152.32 (9)
C18A—C13A—C14A—C15A	−70.58 (11)	C15C—C16C—C17C—C13C	22.60 (10)
C17A—C13A—C14A—C15A	46.82 (9)	C12C—C13C—C17C—C20C	76.31 (12)
C8A—C14A—C15A—C16A	−159.83 (8)	C18C—C13C—C17C—C20C	−48.34 (15)
C13A—C14A—C15A—C16A	−33.10 (9)	C14C—C13C—C17C—C20C	−167.40 (10)
C14A—C15A—C16A—C17A	6.74 (10)	C12C—C13C—C17C—C16C	−158.26 (8)
C12A—C13A—C17A—C20A	76.84 (10)	C18C—C13C—C17C—C16C	77.10 (12)
C18A—C13A—C17A—C20A	−47.63 (13)	C14C—C13C—C17C—C16C	−41.97 (9)
C14A—C13A—C17A—C20A	−166.76 (8)	C16C—C17C—C20C—C21C	−177.99 (10)
C12A—C13A—C17A—C16A	−157.94 (8)	C13C—C17C—C20C—C21C	−57.24 (14)
C18A—C13A—C17A—C16A	77.59 (11)	C16C—C17C—C20C—C22C	57.03 (13)
C14A—C13A—C17A—C16A	−41.54 (8)	C13C—C17C—C20C—C22C	177.78 (10)
C15A—C16A—C17A—C20A	151.32 (8)	C21C—C20C—C22C—C23C	−64.57 (16)
C15A—C16A—C17A—C13A	21.94 (9)	C17C—C20C—C22C—C23C	62.58 (17)
C13A—C17A—C20A—C21A	−49.93 (13)	C20C—C22C—C23C—C24C	179.60 (12)
C16A—C17A—C20A—C21A	−170.38 (9)	C22C—C23C—C24C—S25C	−173.31 (10)
C13A—C17A—C20A—C22A	−174.25 (9)	C23C—C24C—S25C—S25D	−72.05 (11)
C16A—C17A—C20A—C22A	65.29 (11)	C4C—C3C—O31C—C32C	144.61 (10)
C21A—C20A—C22A—C23A	−64.36 (14)	C2C—C3C—O31C—C32C	−95.97 (12)
C17A—C20A—C22A—C23A	62.96 (13)	C3C—O31C—C32C—O33C	−5.86 (19)
C20A—C22A—C23A—C24A	165.22 (10)	C3C—O31C—C32C—C34C	172.24 (9)
C22A—C23A—C24A—S25A	69.24 (14)	O33C—C32C—C34C—C35C	3.94 (16)
C23A—C24A—S25A—S25B	63.65 (9)	O31C—C32C—C34C—C35C	−174.23 (10)
C4A—C3A—O31A—C32A	163.68 (8)	O33C—C32C—C34C—C39C	−179.61 (12)
C2A—C3A—O31A—C32A	−75.28 (12)	O31C—C32C—C34C—C39C	2.22 (14)
C3A—O31A—C32A—O33A	−0.33 (15)	C39C—C34C—C35C—C36C	−0.61 (15)
C3A—O31A—C32A—C34A	−179.78 (8)	C32C—C34C—C35C—C36C	175.90 (10)
O33A—C32A—C34A—C35A	1.92 (15)	C34C—C35C—C36C—C37C	−0.10 (16)
O31A—C32A—C34A—C35A	−178.61 (9)	C35C—C36C—C37C—C38C	0.74 (15)
O33A—C32A—C34A—C39A	−177.91 (10)	C35C—C36C—C37C—N40C	−178.18 (9)
O31A—C32A—C34A—C39A	1.55 (13)	C36C—C37C—C38C—C39C	−0.64 (15)
C39A—C34A—C35A—C36A	−0.93 (14)	N40C—C37C—C38C—C39C	178.29 (9)
C32A—C34A—C35A—C36A	179.23 (9)	C37C—C38C—C39C—C34C	−0.10 (14)
C34A—C35A—C36A—C37A	−0.27 (14)	C35C—C34C—C39C—C38C	0.71 (15)
C35A—C36A—C37A—C38A	1.19 (15)	C32C—C34C—C39C—C38C	−175.67 (9)
C35A—C36A—C37A—N40A	−178.44 (8)	C36C—C37C—N40C—O41C	−10.91 (15)
C36A—C37A—C38A—C39A	−0.86 (15)	C38C—C37C—N40C—O41C	170.11 (11)
N40A—C37A—C38A—C39A	178.76 (9)	C36C—C37C—N40C—O42C	168.35 (10)
C37A—C38A—C39A—C34A	−0.38 (14)	C38C—C37C—N40C—O42C	−10.63 (14)
C35A—C34A—C39A—C38A	1.26 (14)	C13C—C12C—O51C—C52C	−151.96 (9)
C32A—C34A—C39A—C38A	−178.91 (9)	C11C—C12C—O51C—C52C	85.18 (11)
C36A—C37A—N40A—O41A	−15.91 (14)	C12C—O51C—C52C—O53C	−5.31 (17)
C38A—C37A—N40A—O41A	164.44 (10)	C12C—O51C—C52C—C54C	174.61 (8)
C36A—C37A—N40A—O42A	163.12 (10)	O53C—C52C—C54C—C59C	158.18 (13)
C38A—C37A—N40A—O42A	−16.53 (14)	O51C—C52C—C54C—C59C	−21.74 (14)
C11A—C12A—O51A—C52A	83.70 (10)	O53C—C52C—C54C—C55C	−21.14 (17)
C13A—C12A—O51A—C52A	−153.58 (8)	O51C—C52C—C54C—C55C	158.94 (9)
C12A—O51A—C52A—O53A	−7.57 (15)	C59C—C54C—C55C—C56C	2.87 (15)
C12A—O51A—C52A—C54A	171.49 (7)	C52C—C54C—C55C—C56C	−177.80 (9)
O53A—C52A—C54A—C55A	−12.34 (18)	C54C—C55C—C56C—C57C	−0.95 (14)
O51A—C52A—C54A—C55A	168.58 (11)	C55C—C56C—C57C—C58C	−2.33 (14)
O53A—C52A—C54A—C59A	166.35 (12)	C55C—C56C—C57C—N60C	174.14 (8)
O51A—C52A—C54A—C59A	−12.73 (14)	C56C—C57C—C58C—C59C	3.61 (14)
C59A—C54A—C55A—C56A	3.0 (2)	N60C—C57C—C58C—C59C	−172.86 (8)
C52A—C54A—C55A—C56A	−178.32 (14)	C57C—C58C—C59C—C54C	−1.59 (14)
C54A—C55A—C56A—C57A	0.1 (2)	C55C—C54C—C59C—C58C	−1.57 (14)
C55A—C56A—C57A—C58A	−4.3 (2)	C52C—C54C—C59C—C58C	179.13 (9)
C55A—C56A—C57A—N60A	178.86 (14)	C58C—C57C—N60C—O61C	−173.16 (10)
C56A—C57A—C58A—C59A	5.28 (19)	C56C—C57C—N60C—O61C	10.23 (13)
N60A—C57A—C58A—C59A	−177.82 (11)	C58C—C57C—N60C—O62C	9.03 (12)
C57A—C58A—C59A—C54A	−2.07 (18)	C56C—C57C—N60C—O62C	−167.59 (9)
C55A—C54A—C59A—C58A	−1.94 (18)	C6C—C7C—O71C—C72C	−104.44 (10)
C52A—C54A—C59A—C58A	179.38 (11)	C8C—C7C—O71C—C72C	133.07 (9)
C56A—C57A—N60A—C62A	−155.35 (16)	C7C—O71C—C72C—O73C	−2.43 (15)
C58A—C57A—N60A—C62A	27.67 (19)	C7C—O71C—C72C—C74C	177.44 (8)
C56A—C57A—N60A—O61A	25.27 (18)	O73C—C72C—C74C—C75C	−5.82 (15)
C58A—C57A—N60A—O61A	−151.71 (13)	O71C—C72C—C74C—C75C	174.30 (9)
C6A—C7A—O71A—C72A	−78.51 (11)	O73C—C72C—C74C—C79C	174.24 (11)
C8A—C7A—O71A—C72A	157.32 (8)	O71C—C72C—C74C—C79C	−5.64 (13)
C7A—O71A—C72A—O73A	−0.85 (15)	C79C—C74C—C75C—C76C	1.06 (17)
C7A—O71A—C72A—C74A	−179.48 (8)	C72C—C74C—C75C—C76C	−178.88 (10)
O73A—C72A—C74A—C79A	−173.45 (11)	C74C—C75C—C76C—C77C	0.27 (17)
O71A—C72A—C74A—C79A	5.21 (14)	C75C—C76C—C77C—C78C	−1.90 (18)
O73A—C72A—C74A—C75A	4.20 (16)	C75C—C76C—C77C—N80C	175.25 (10)
O71A—C72A—C74A—C75A	−177.14 (9)	C76C—C77C—C78C—C79C	2.13 (17)
C79A—C74A—C75A—C76A	1.83 (17)	N80C—C77C—C78C—C79C	−175.04 (10)
C72A—C74A—C75A—C76A	−175.87 (10)	C77C—C78C—C79C—C74C	−0.72 (17)
C74A—C75A—C76A—C77A	1.01 (17)	C75C—C74C—C79C—C78C	−0.83 (16)
C75A—C76A—C77A—C78A	−2.97 (16)	C72C—C74C—C79C—C78C	179.11 (10)
C75A—C76A—C77A—N80A	175.20 (10)	C78C—C77C—N80C—O81C	−169.20 (14)
C76A—C77A—C78A—C79A	2.02 (16)	C76C—C77C—N80C—O81C	13.51 (17)
N80A—C77A—C78A—C79A	−176.16 (9)	C78C—C77C—N80C—O82C	11.95 (15)
C75A—C74A—C79A—C78A	−2.79 (15)	C76C—C77C—N80C—O82C	−165.34 (12)
C72A—C74A—C79A—C78A	174.77 (9)	C10D—C1D—C2D—C3D	57.80 (12)
C77A—C78A—C79A—C74A	0.92 (15)	C1D—C2D—C3D—O31D	−175.17 (8)
C78A—C77A—N80A—O81A	−168.93 (11)	C1D—C2D—C3D—C4D	−58.97 (12)
C76A—C77A—N80A—O81A	12.82 (16)	O31D—C3D—C4D—C5D	177.85 (8)
C78A—C77A—N80A—C82A	11.40 (16)	C2D—C3D—C4D—C5D	58.64 (12)
C76A—C77A—N80A—C82A	−166.85 (11)	C3D—C4D—C5D—C6D	177.62 (8)
C10B—C1B—C2B—C3B	58.60 (11)	C3D—C4D—C5D—C10D	−54.79 (12)
C1B—C2B—C3B—O31B	−173.44 (7)	C4D—C5D—C6D—C7D	77.67 (12)
C1B—C2B—C3B—C4B	−57.02 (11)	C10D—C5D—C6D—C7D	−50.38 (11)
O31B—C3B—C4B—C5B	176.36 (7)	C5D—C6D—C7D—O71D	−70.22 (11)
C2B—C3B—C4B—C5B	56.33 (12)	C5D—C6D—C7D—C8D	48.73 (11)
C3B—C4B—C5B—C6B	179.27 (7)	O71D—C7D—C8D—C14D	−55.35 (11)
C3B—C4B—C5B—C10B	−53.93 (11)	C6D—C7D—C8D—C14D	−176.57 (7)
C4B—C5B—C6B—C7B	77.73 (11)	O71D—C7D—C8D—C9D	69.15 (11)
C10B—C5B—C6B—C7B	−49.52 (10)	C6D—C7D—C8D—C9D	−52.07 (10)
C5B—C6B—C7B—O71B	−72.84 (11)	C14D—C8D—C9D—C11D	−50.89 (9)
C5B—C6B—C7B—C8B	46.77 (11)	C7D—C8D—C9D—C11D	−176.41 (7)
O71B—C7B—C8B—C14B	−51.97 (9)	C14D—C8D—C9D—C10D	−177.17 (7)
C6B—C7B—C8B—C14B	−173.76 (7)	C7D—C8D—C9D—C10D	57.32 (9)
O71B—C7B—C8B—C9B	71.47 (11)	C2D—C1D—C10D—C19D	−169.27 (8)
C6B—C7B—C8B—C9B	−50.32 (10)	C2D—C1D—C10D—C5D	−52.91 (11)
C14B—C8B—C9B—C11B	−50.47 (9)	C2D—C1D—C10D—C9D	68.34 (13)
C7B—C8B—C9B—C11B	−174.75 (7)	C6D—C5D—C10D—C19D	−68.21 (12)
C14B—C8B—C9B—C10B	−177.77 (7)	C4D—C5D—C10D—C19D	164.80 (9)
C7B—C8B—C9B—C10B	57.96 (9)	C6D—C5D—C10D—C1D	177.31 (7)
C2B—C1B—C10B—C19B	−172.45 (8)	C4D—C5D—C10D—C1D	50.33 (12)
C2B—C1B—C10B—C5B	−55.03 (11)	C6D—C5D—C10D—C9D	53.83 (10)
C2B—C1B—C10B—C9B	64.92 (11)	C4D—C5D—C10D—C9D	−73.16 (11)
C6B—C5B—C10B—C1B	177.83 (7)	C11D—C9D—C10D—C19D	−62.23 (12)
C4B—C5B—C10B—C1B	51.21 (11)	C8D—C9D—C10D—C19D	62.94 (11)
C6B—C5B—C10B—C19B	−67.24 (11)	C11D—C9D—C10D—C1D	56.93 (10)
C4B—C5B—C10B—C19B	166.15 (8)	C8D—C9D—C10D—C1D	−177.90 (7)
C6B—C5B—C10B—C9B	55.37 (10)	C11D—C9D—C10D—C5D	177.43 (7)
C4B—C5B—C10B—C9B	−71.25 (9)	C8D—C9D—C10D—C5D	−57.40 (9)
C11B—C9B—C10B—C1B	53.90 (10)	C8D—C9D—C11D—C12D	48.16 (9)
C8B—C9B—C10B—C1B	−179.73 (7)	C10D—C9D—C11D—C12D	173.23 (7)
C11B—C9B—C10B—C19B	−65.34 (12)	C9D—C11D—C12D—O51D	67.55 (11)
C8B—C9B—C10B—C19B	61.02 (10)	C9D—C11D—C12D—C13D	−51.19 (10)
C11B—C9B—C10B—C5B	173.35 (7)	O51D—C12D—C13D—C18D	173.58 (7)
C8B—C9B—C10B—C5B	−60.29 (9)	C11D—C12D—C13D—C18D	−67.24 (11)
C8B—C9B—C11B—C12B	48.64 (10)	O51D—C12D—C13D—C14D	−64.54 (9)
C10B—C9B—C11B—C12B	174.61 (7)	C11D—C12D—C13D—C14D	54.65 (9)
C9B—C11B—C12B—O51B	68.53 (12)	O51D—C12D—C13D—C17D	47.84 (11)
C9B—C11B—C12B—C13B	−51.16 (10)	C11D—C12D—C13D—C17D	167.02 (7)
O51B—C12B—C13B—C18B	170.25 (7)	C7D—C8D—C14D—C15D	−52.90 (10)
C11B—C12B—C13B—C18B	−69.04 (10)	C9D—C8D—C14D—C15D	−178.50 (7)
O51B—C12B—C13B—C14B	−67.15 (10)	C7D—C8D—C14D—C13D	−174.34 (7)
C11B—C12B—C13B—C14B	53.56 (9)	C9D—C8D—C14D—C13D	60.05 (9)
O51B—C12B—C13B—C17B	44.73 (10)	C12D—C13D—C14D—C8D	−61.18 (9)
C11B—C12B—C13B—C17B	165.44 (7)	C18D—C13D—C14D—C8D	57.99 (11)
C7B—C8B—C14B—C15B	−55.40 (10)	C17D—C13D—C14D—C8D	174.86 (7)
C9B—C8B—C14B—C15B	−179.30 (7)	C12D—C13D—C14D—C15D	170.14 (7)
C7B—C8B—C14B—C13B	−178.15 (7)	C18D—C13D—C14D—C15D	−70.70 (11)
C9B—C8B—C14B—C13B	57.95 (9)	C17D—C13D—C14D—C15D	46.17 (8)
C12B—C13B—C14B—C8B	−58.94 (10)	C8D—C14D—C15D—C16D	−159.08 (8)
C18B—C13B—C14B—C8B	60.41 (10)	C13D—C14D—C15D—C16D	−33.09 (10)
C17B—C13B—C14B—C8B	177.22 (7)	C14D—C15D—C16D—C17D	6.91 (10)
C12B—C13B—C14B—C15B	170.94 (7)	C15D—C16D—C17D—C20D	149.46 (8)
C18B—C13B—C14B—C15B	−69.71 (11)	C15D—C16D—C17D—C13D	21.27 (9)
C17B—C13B—C14B—C15B	47.10 (9)	C12D—C13D—C17D—C20D	79.71 (10)
C8B—C14B—C15B—C16B	−159.84 (8)	C18D—C13D—C17D—C20D	−45.20 (11)
C13B—C14B—C15B—C16B	−32.35 (9)	C14D—C13D—C17D—C20D	−164.01 (8)
C14B—C15B—C16B—C17B	4.72 (9)	C12D—C13D—C17D—C16D	−156.88 (7)
C15B—C16B—C17B—C20B	153.19 (8)	C18D—C13D—C17D—C16D	78.21 (11)
C15B—C16B—C17B—C13B	24.10 (9)	C14D—C13D—C17D—C16D	−40.60 (8)
C12B—C13B—C17B—C20B	75.80 (10)	C16D—C17D—C20D—C21D	−178.10 (8)
C18B—C13B—C17B—C20B	−48.63 (12)	C13D—C17D—C20D—C21D	−59.03 (11)
C14B—C13B—C17B—C20B	−167.65 (8)	C16D—C17D—C20D—C22D	56.45 (11)
C12B—C13B—C17B—C16B	−159.41 (7)	C13D—C17D—C20D—C22D	175.52 (8)
C18B—C13B—C17B—C16B	76.17 (10)	C21D—C20D—C22D—C23D	−60.58 (14)
C14B—C13B—C17B—C16B	−42.85 (8)	C17D—C20D—C22D—C23D	66.30 (13)
C16B—C17B—C20B—C21B	178.54 (8)	C20D—C22D—C23D—C24D	−177.03 (11)
C13B—C17B—C20B—C21B	−61.53 (11)	C22D—C23D—C24D—S25D	−170.50 (9)
C16B—C17B—C20B—C22B	53.94 (11)	C23D—C24D—S25D—S25C	−68.36 (12)
C13B—C17B—C20B—C22B	173.88 (8)	C24C—S25C—S25D—C24D	−92.50 (10)
C21B—C20B—C22B—C23B	−65.69 (12)	C2D—C3D—O31D—C32D	−97.75 (12)
C17B—C20B—C22B—C23B	60.06 (13)	C4D—C3D—O31D—C32D	142.29 (10)
C20B—C22B—C23B—C24B	173.02 (9)	C3D—O31D—C32D—O33D	2.95 (19)
C22B—C23B—C24B—S25B	69.73 (13)	C3D—O31D—C32D—C34D	−176.98 (9)
C23B—C24B—S25B—S25A	67.46 (9)	O33D—C32D—C34D—C35D	8.64 (18)
C24A—S25A—S25B—C24B	70.94 (9)	O31D—C32D—C34D—C35D	−171.42 (10)
C2B—C3B—O31B—C32B	−72.97 (12)	O33D—C32D—C34D—C39D	−171.73 (13)
C4B—C3B—O31B—C32B	166.75 (8)	O31D—C32D—C34D—C39D	8.20 (14)
C3B—O31B—C32B—O33B	−3.40 (16)	C39D—C34D—C35D—C36D	−0.89 (16)
C3B—O31B—C32B—C34B	176.83 (8)	C32D—C34D—C35D—C36D	178.74 (10)
O33B—C32B—C34B—C35B	14.31 (15)	C34D—C35D—C36D—C37D	−0.22 (17)
O31B—C32B—C34B—C35B	−165.91 (9)	C35D—C36D—C37D—C38D	1.11 (16)
O33B—C32B—C34B—C39B	−164.72 (11)	C35D—C36D—C37D—N40D	−178.56 (10)
O31B—C32B—C34B—C39B	15.06 (13)	C36D—C37D—C38D—C39D	−0.85 (15)
C39B—C34B—C35B—C36B	−0.33 (16)	N40D—C37D—C38D—C39D	178.82 (9)
C32B—C34B—C35B—C36B	−179.38 (9)	C37D—C38D—C39D—C34D	−0.31 (15)
C34B—C35B—C36B—C37B	0.38 (16)	C35D—C34D—C39D—C38D	1.16 (15)
C35B—C36B—C37B—C38B	−0.20 (16)	C32D—C34D—C39D—C38D	−178.45 (10)
C35B—C36B—C37B—N40B	179.58 (9)	C36D—C37D—N40D—O41D	−22.29 (17)
C36B—C37B—C38B—C39B	−0.03 (17)	C38D—C37D—N40D—O41D	158.02 (12)
N40B—C37B—C38B—C39B	−179.81 (10)	C36D—C37D—N40D—O42D	158.71 (12)
C37B—C38B—C39B—C34B	0.08 (18)	C38D—C37D—N40D—O42D	−20.98 (16)
C35B—C34B—C39B—C38B	0.10 (16)	C11D—C12D—O51D—C52D	84.22 (10)
C32B—C34B—C39B—C38B	179.11 (10)	C13D—C12D—O51D—C52D	−154.48 (8)
C36B—C37B—N40B—O41B	2.84 (15)	C12D—O51D—C52D—O53D	2.82 (17)
C38B—C37B—N40B—O41B	−177.37 (11)	C12D—O51D—C52D—C54D	−176.12 (7)
C36B—C37B—N40B—O42B	−176.30 (15)	O53D—C52D—C54D—C55D	1.71 (17)
C38B—C37B—N40B—O42B	3.50 (18)	O51D—C52D—C54D—C55D	−179.33 (9)
C11B—C12B—O51B—C52B	86.46 (10)	O53D—C52D—C54D—C59D	−177.41 (12)
C13B—C12B—O51B—C52B	−151.01 (8)	O51D—C52D—C54D—C59D	1.54 (13)
C12B—O51B—C52B—O53B	−5.50 (15)	C59D—C54D—C55D—C56D	−0.16 (15)
C12B—O51B—C52B—C54B	173.15 (7)	C52D—C54D—C55D—C56D	−179.29 (10)
O53B—C52B—C54B—C59B	−176.07 (11)	C54D—C55D—C56D—C57D	−0.10 (15)
O51B—C52B—C54B—C59B	5.25 (13)	C55D—C56D—C57D—C58D	0.65 (15)
O53B—C52B—C54B—C55B	5.30 (16)	C55D—C56D—C57D—N60D	−179.55 (9)
O51B—C52B—C54B—C55B	−173.37 (9)	C56D—C57D—C58D—C59D	−0.92 (14)
C59B—C54B—C55B—C56B	−1.77 (17)	N60D—C57D—C58D—C59D	179.28 (8)
C52B—C54B—C55B—C56B	176.87 (10)	C57D—C58D—C59D—C54D	0.62 (13)
C54B—C55B—C56B—C57B	0.95 (18)	C55D—C54D—C59D—C58D	−0.11 (14)
C55B—C56B—C57B—C58B	1.29 (17)	C52D—C54D—C59D—C58D	178.99 (9)
C55B—C56B—C57B—N60B	−177.20 (10)	C56D—C57D—N60D—O61D	−8.63 (14)
C56B—C57B—C58B—C59B	−2.65 (15)	C58D—C57D—N60D—O61D	171.17 (10)
N60B—C57B—C58B—C59B	175.86 (9)	C56D—C57D—N60D—O62D	172.00 (10)
C55B—C54B—C59B—C58B	0.39 (15)	C58D—C57D—N60D—O62D	−8.20 (13)
C52B—C54B—C59B—C58B	−178.20 (9)	C6D—C7D—O71D—C72D	−95.17 (11)
C57B—C58B—C59B—C54B	1.76 (14)	C8D—C7D—O71D—C72D	142.45 (8)
C56B—C57B—N60B—O61B	8.68 (17)	C7D—O71D—C72D—O73D	3.00 (14)
C58B—C57B—N60B—O61B	−169.89 (13)	C7D—O71D—C72D—C74D	−175.25 (7)
C56B—C57B—N60B—O62B	−172.58 (12)	O73D—C72D—C74D—C75D	−3.39 (14)
C58B—C57B—N60B—O62B	8.85 (15)	O71D—C72D—C74D—C75D	174.89 (9)
C6B—C7B—O71B—C72B	−89.95 (10)	O73D—C72D—C74D—C79D	178.07 (10)
C8B—C7B—O71B—C72B	146.20 (8)	O71D—C72D—C74D—C79D	−3.65 (12)
C7B—O71B—C72B—O73B	4.09 (13)	C79D—C74D—C75D—C76D	1.11 (15)
C7B—O71B—C72B—C74B	−175.60 (7)	C72D—C74D—C75D—C76D	−177.45 (9)
O73B—C72B—C74B—C75B	7.83 (14)	C74D—C75D—C76D—C77D	−1.58 (16)
O71B—C72B—C74B—C75B	−172.48 (8)	C75D—C76D—C77D—C78D	0.78 (16)
O73B—C72B—C74B—C79B	−171.76 (9)	C75D—C76D—C77D—N80D	179.98 (10)
O71B—C72B—C74B—C79B	7.94 (12)	C76D—C77D—C78D—C79D	0.51 (16)
C79B—C74B—C75B—C76B	−0.09 (14)	N80D—C77D—C78D—C79D	−178.70 (9)
C72B—C74B—C75B—C76B	−179.68 (9)	C77D—C78D—C79D—C74D	−1.00 (15)
C74B—C75B—C76B—C77B	0.15 (15)	C75D—C74D—C79D—C78D	0.22 (15)
C75B—C76B—C77B—C78B	0.25 (15)	C72D—C74D—C79D—C78D	178.73 (9)
C75B—C76B—C77B—N80B	179.96 (9)	C76D—C77D—N80D—O81D	−10.08 (15)
C76B—C77B—C78B—C79B	−0.70 (14)	C78D—C77D—N80D—O81D	169.16 (10)
N80B—C77B—C78B—C79B	179.58 (8)	C76D—C77D—N80D—O82D	170.88 (11)
C77B—C78B—C79B—C74B	0.75 (13)	C78D—C77D—N80D—O82D	−9.88 (15)
C75B—C74B—C79B—C78B	−0.37 (13)	O103—C102—O104—C105	−4.9 (2)
C72B—C74B—C79B—C78B	179.21 (8)	C101—C102—O104—C105	177.15 (11)
C76B—C77B—N80B—O81B	−11.44 (14)	C102—O104—C105—C106	−147.30 (13)
C78B—C77B—N80B—O81B	168.28 (10)	O203—C202—O204—C205	15.3 (4)
C76B—C77B—N80B—O82B	169.05 (10)	C201—C202—O204—C205	−165.4 (4)
C78B—C77B—N80B—O82B	−11.22 (14)	C202—O204—C205—C206	−169.5 (3)
C10C—C1C—C2C—C3C	57.56 (12)	O213—C212—O214—C215	6.4 (4)
C1C—C2C—C3C—O31C	−171.71 (8)	C211—C212—O214—C215	−174.0 (3)
C1C—C2C—C3C—C4C	−56.22 (12)	C212—O214—C215—C216	105.6 (5)
O31C—C3C—C4C—C5C	173.16 (8)	C301—C302—C303—C304	−172.4 (5)
C2C—C3C—C4C—C5C	55.95 (12)	C302—C303—C304—C305	−171.8 (5)
C3C—C4C—C5C—C6C	178.44 (8)	C303—C304—C305—C306	−176.7 (5)
